# Biodegradable Tea Saponin/Xylan/PVA Composite Films with Antibacterial and Antioxidant Properties for Strawberry Preservation

**DOI:** 10.3390/foods15193421

**Published:** 2026-09-24

**Authors:** Yi-Heng Xiao, Juan-Juan Jiang, Jin-Yang Wang, Cheng-Liang Wu, Jia-Dong Guo, Qiang Li

**Affiliations:** 1 Department of Chemistry, College of Science, Beijing Forestry University, Beijing 100083, China; 2 College of Environmental Science & Engineering, Beijing Forestry University, Beijing 100083, China; 3 Department of Economics and Management, Beijing Forestry University, Beijing 100083, China

**Keywords:** composite films, tea saponin, xylan, biodegradable packaging, strawberry preservation

## Abstract

Conventional non-degradable plastic films used in postharvest packaging cause environmental pollution and offer limited functionality for extending shelf life. The booming *Camellia oleifera* oil industry generates massive tea shell waste, whose improper disposal is both a resource loss and an opportunity for developing biodegradable active packaging. Here, alkali-extracted xylan (AX) and tea saponin (TS) were blended with PVA to prepare TS/AX/PVA composite films. The optimized films exhibited low moisture content (10.39%), reduced water solubility (0.35%), enhanced UV-blocking and water vapor-barrier properties, near-complete visual disintegration within 30 days of soil burial (visual soil-burial assessment only), and elongation at break up to 330.81%. The incorporation of AX improved the mechanical strength and degradability of the PVA matrix, while TS imparted antioxidant and antibacterial activities, yielding better hydrophobicity, higher tensile strength, and enhanced antibacterial performance than neat PVA. In fresh strawberry preservation, the 6% TS/AX/PVA film retarded quality deterioration, maintaining 18.43% lower relative conductivity than the control, 0.51% titratable acidity, and 26.64 mg/100 g vitamin C (71.21% higher than the PE group) on day 10. This study converts agricultural by-products into biodegradable functional films and suggests a plausible, but not directly measured, preservation mechanism that requires verification, offering a promising substitute for conventional packaging plastics.

## 1. Introduction

Microbial contamination, moisture ingress, oxygen permeation, and light exposure are widely recognized as primary abiotic stressors driving food-quality deterioration [1]. These postharvest processes not only compromise the nutritional density and sensory profile of fresh produce but also lead to the formation of hazardous metabolites, thereby posing significant risks to consumer health. To mitigate the deleterious effects of these postharvest environmental factors and establish a robust food safety assurance system throughout the postharvest supply chain, advanced food-packaging materials with functional protective capabilities have emerged as a key research priority in postharvest science and engineering [2]. Currently, petroleum-derived polymers, including polyesters, polyolefins, and polyvinyl alcohol (PVA), dominate the global food-packaging market due to their low cost, excellent mechanical strength, and favorable processability [3,4]. Although PVA is a water-soluble synthetic polymer whose biodegradation by specific enzyme consortia and appropriate microbial conditions is well documented, its degradation in natural soil is relatively slow and largely dependent on acclimated microorganisms. The inherent recalcitrance of these PVA materials to biodegradation, compounded by inadequate waste management infrastructure, has precipitated a global ecological crisis and raised increasing concerns about long-term public health implications [5]. In response to these challenges, the development of sustainable, non-toxic, biodegradable, and easy-to-process alternative materials has become an urgent imperative for the food industry to transition toward a circular economy.

As the market demand for *Camellia oleifera* oil rapidly expands, large quantities of its husks are typically discarded via random stockpiling or incineration [6]. This leads to considerable resource waste and environmental pollution. Prior research has predominantly focused on extracting bioactive components, such as saponins and polysaccharides; however, the development of functional preservation films using tea saponin (TS) as a natural antibacterial agent and high-barrier xylan derived from *Camellia oleifera* husks has not yet been reported [7]. Xylan, an abundant and renewable biopolymer, exhibits strong film-forming properties, biodegradability, and inherent gas-barrier capabilities; thus, it is a promising matrix material for functional and environmentally sustainable food-packaging films [8]. With appropriate modifications or composite strategies, xylan-based films can achieve antimicrobial, antioxidant, and ultraviolet (UV) light-blocking functions, extending the shelf life of packaged foods [9]. Prior studies have demonstrated that TS, a natural biosurfactant, can be incorporated into various biopolymer matrices to produce active food-packaging films with multiple benefits. For example, TS-functionalized chitosan films offer antimicrobial and antioxidant properties, and TS-modified poly(lactic acid) composite films improve antibacterial and antioxidant performance for meat preservation. TS-based emulsified edible films have also been developed as active packaging for oil-rich foods, providing barrier and oxidative stability through emulsification [10]. These applications highlight TS’s versatility as a bioactive ingredient for next-generation active packaging. *Camellia oleifera* husks, as abundant, renewable, and low-cost agroforestry by-products, are ideal feedstocks for eco-friendly food-packaging materials. From a packaging-design perspective, incorporating TS provides inherent antibacterial activity. Additionally, the dense hydrogen-bond network formed by hydroxyl groups endows xylan with remarkable biocompatibility and biodegradability. Although its oxygen-barrier performance is typically inferior to that of conventional polyethylene (PE) films under humid conditions, it can be substantially improved through chemical modifications or composite strategies [11,12].

Xylan-based films are inherently brittle and highly moisture-sensitive. Water absorption easily disrupts their internal structure, leading to major deterioration in mechanical and barrier performance. Common strategies to address these limitations include reinforcement, plasticization, and chemical modification [13]. Simsek et al. extracted arabinoxylan from corn processing by-products and developed sustainable food-packaging films via lipase–acetate surface modification [14]. This process notably enhanced film flexibility, tensile strength, and tear resistance, with films derived from dry-milled corn bran exhibiting superior overall performance. Structural analysis confirmed the presence of ester groups, which improved hydrophobicity without compromising biodegradability. Weng et al. incorporated nano-ZnO into a xylan/PVA matrix using sodium hexametaphosphate as a dispersant. At an optimal 2 wt% loading, the composite film demonstrated robust performance, including enhanced mechanical strength, improved oxygen and water vapor-barrier properties, higher thermal stability, and greater surface hydrophobicity. Peng et al. prepared xylan-based films via sodium periodate oxidation, followed by solution casting, revealing that aldehyde content influences the films’ mechanical properties, transparency, and hydrophobicity.

The present study investigates the efficient separation and purification of TS and xylan from waste *Camellia oleifera* husks through a cascade-extraction approach that combines ultrasound-assisted hydrothermal treatment with sequential acid–base purification. Furthermore, hydrogen peroxide and D151 + D301 composite resins were used to optimize the decolorization and purification of these renewable components, with their fundamental physicochemical properties systematically characterized. Subsequently, alkali-extracted xylan (AX) was blended with PVA to form a film matrix, with TS isolated from the same biomass introduced as an antioxidant and antibacterial agent. The TS/AX/PVA films were fabricated via solution casting. This integrated strategy effectively overcomes the limitations of neat PVA films, enhances the composite’s biodegradability, and promotes high-value utilization of postharvest-waste *Camellia oleifera* husks. The resulting composites exhibit improved mechanical strength, lower water solubility, and reliable antibacterial and preservation functions, making them a promising eco-friendly material for prolonging the postharvest shelf life of perishable products, such as strawberries.

## 2. Materials and Methods

### 2.1. Materials

In this experiment, the TS and xylan utilized were extracted from the processing waste of *Camellia oleifera* (i.e., *camellia* seed shells). We optimized the ultrasound-assisted aqueous extraction of TS from *Camellia oleifera* husks, as well as the subsequent hydrogen-peroxide decolorization procedure (Supplementary Data S1). Subsequently, xylan was isolated from the post-extraction *Camellia oleifera* husk residue via a combined acid–alkali extraction process. Furthermore, we optimized the xylan extraction process and established a combined decolorization and desalination protocol employing anion–cation exchange resins (Supplementary Data S3). PVA (type 1788), glycerol, 2,6-dichloroindophenol, standard ascorbic acid (vitamin C, VitC), phenolphthalein, and anhydrous calcium chloride were purchased from Macklin Biochemical Technology Co., Ltd. (Shanghai, China). Luria-Bertani (LB) media was obtained from Beijing AuBoStar Biotechnology Co., Ltd. (Beijing, China). DPPH was purchased from Sigma-Aldrich Chemical Company (St. Louis, MO, USA). Oxalic acid and sodium hydroxide were acquired from Beijing Chemical Factory (Beijing, China). Fresh Dandong strawberries were procured from a local supermarket. All chemicals were of analytical grade and used without further purification.

### 2.2. Fabrication and Mechanical Tests of AX/PVA Films

Preparation of 4% (*w*/*v*) PVA solution: PVA (4 g) was dissolved in distilled water (100 mL) under continuous stirring at 90 °C to form a homogeneous, transparent solution. Preparation of 4% (*w*/*v*) AX solution: Freeze-dried AX powder (2 g) was ground in a mortar and dissolved in distilled water (50 mL) under stirring at 60 °C to obtain a clear solution. Film-forming solutions (20 mL) were prepared by mixing 4% AX and 4% PVA solutions at various volume ratios (1:9, 2:8, 3:7, 4:6, and 5:5), with glycerol added at 10% of the total solid mass of PVA and AX. The mixtures were ultrasonicated for 10 min, then stirred at 75 °C for 1 h, cast onto glass plates, and dried at 50 °C to obtain composite films with different AX/PVA ratios. Film thickness was measured at five random positions using a digital micrometer (0–150 mm, Mitutoyo, Kawasaki, Japan), and the average value was calculated. Tensile properties and elongation at break were tested using an electronic universal testing machine (Model CMT6103, MIS Industrial Systems Co., Ltd., Rochester Hills, MI, USA) to characterize the mechanical performance.

### 2.3. Fabrication of TS-Incorporated AX/PVA Films

A 4% (*w*/*v*) PVA solution and a 4% (*w*/*v*) AX solution were mixed at a predetermined ratio, with stirring at 400 rpm. Glycerol was added at 10% of the total solid mass of PVA and AX. The mixture was ultrasonicated for 10 min and then stirred at 75 °C for 1 h to obtain 60 mL of AX/PVA film-forming solution. Various amounts of TS were added and stirred at 400 rpm for 1 h, followed by ultrasonication for 10 min to remove air bubbles. Films were conditioned in a desiccator at 25 ± 1 °C, 52 ± 3% relative humidity, and in the dark prior to testing. The film with 2.4 g TS showed a final concentration of 4% (*w*/*v*) and was designated as 4% TS/AX/PVA. Similarly, films containing 3.6 g and 4.8 g TS corresponded to 6% and 8% (*w*/*v*) TS contents, labeled as 6% TS/AX/PVA and 8% TS/AX/PVA, respectively.

### 2.4. Characterization of TS/AX/PVA Films

#### 2.4.1. Morphological Characteristics

The macroscopic morphology of the composite film was recorded using a smartphone camera.

#### 2.4.2. Thickness

Film thickness (d, mm) was measured at five random positions on each specimen using an electronic digital micrometer. Each sample was tested in triplicate, and the average value was taken as the final thickness.

#### 2.4.3. Water Content and Water Solubility

Moisture content (MC, %) of composite films was determined by the weight-loss drying method and calculated using Equation (1), where M_1_ (g) is the initial mass of the film, and M_2_ (g) is the mass after drying to constant weight [15].

(1) $$MC(\%)=\frac{M_{1}-M_{2}}{M_{1}}\times 100$$

Water solubility (WS, %) of composite films was determined by the method of Silva et al. and calculated using Equation (2), where W_1_ (g) is the weight of the sample after drying to constant mass, and W_2_ (g) is the weight of the same sample after immersion and re-drying to constant mass [16].

(2) $$WS(\%)=\frac{W_{1}-W_{2}}{W_{1}}\times 100$$

#### 2.4.4. Fourier-Transform Infrared Spectroscopy

FT-IR spectra of the samples were recorded on a Nicolet iS20 spectrometer (Thermo Fisher Scientific, Waltham, MA, USA) in the range of 4000–500 cm^−1^.

#### 2.4.5. Mechanical Properties

Tensile properties of composite film specimens were tested using an electronic universal testing machine, with tensile strength and elongation at break as key parameters. The samples were cut into 1 cm × 5 cm rectangular strips and conditioned at 25 °C and 55% relative humidity for 24 h. Each specimen was tested in triplicate, and the average values of tensile strength and elongation at break were calculated.

#### 2.4.6. Water Vapor-Barrier Properties

The water-vapor transmission rate (WVTR) of the composite film was measured according to Chinese National Standard GB/T 1037-2021 (China) [17].

There are the following (3) mathematical relationships between *WVTR* and *WVP*, where the film thickness is represented by *d* (m) and the difference in water-vapor pressure on both sides of the film is denoted by Δ*P* (Pa).

(3) $$WVP=\frac{WVTR\times d}{\Delta P}$$

#### 2.4.7. Optical Transmittance Properties

Composite films were cut into 4 cm × 1 cm standard specimens and attached to the inner walls of cuvettes. Spectra were scanned over 200–600 nm using an UV-Vis spectrophotometer (U4150, Hitachi, Ltd., Hitachinaka, Japan), with a blank cuvette as reference.

#### 2.4.8. Thermal Stability

The sample was stored in a weighing bottle with calcium chloride at room temperature for 2 d. A 5 mg aliquot was transferred to a crucible, with an empty crucible as a blank. Thermogravimetric analysis (TGA 7300, Hitachi, Japan) was performed from 50 to 600 °C, and mass changes were recorded.

#### 2.4.9. Biodegradability

Composite films were buried in soil to investigate their degradation under natural conditions. This method simulates real-world biodegradation and assesses environmental implications. Six film samples were cut into 3 cm × 3 cm squares and individually placed in containers filled with moist soil. Deionized water was sprayed at 5-day intervals to maintain constant soil moisture, and degradation was recorded photographically.

#### 2.4.10. Antioxidant Activity

The antioxidant capacity of the composite films was evaluated via DPPH radical scavenging activity (RSA%) [18]. Film specimens (1 cm × 2 cm) were immersed in 0.1 mM DPPH–ethanol solution and incubated in the dark at room temperature for 30 min. Absorbance at 517 nm was measured by UV–visible spectrophotometry, with DPPH blank solution as the reference. DPPH radical scavenging activity was calculated using Equation (4).

(4) $$DPPH\;radical\;scavenging\;activity(\%)=\frac{A_{b}-A_{s}}{A_{b}}\times 100$$

#### 2.4.11. Antibacterial Activity

*Escherichia coli* (*E. coli*) (DH5α), a laboratory strain, and *Staphylococcus aureus* (*S. aureus*) (CICC 10384), a generic Gram-positive model, were used for preliminary screening of the films’ antibacterial activity. We acknowledge that neither strain is well aligned with the microorganisms most relevant to postharvest strawberry spoilage, which is visually dominated by mold development, and that no antifungal organism was tested; therefore, the antibacterial results are reported only as a preliminary functional characterization of the films and cannot be extrapolated to antifungal or strawberry-decay activity. Bacterial suspensions were diluted to 10^4^–10^5^ CFU/mL. Pre-sterilized (UV-treated) composite film samples (25 mm in diameter) were immersed in 5 mL of the bacterial suspension and incubated at 37 °C for 90 min. Subsequently, 0.1 mL of the suspension was evenly spread onto blank LB plates and incubated at 37 °C for 24 h [19]. The inhibitory rate (IR, %) against *E. coli* and *S. aureus* was calculated according to Equations (5) and (6) shown below, where I_1_ and I_2_ are the *E. coli* counts in the control and film sample treatment groups, respectively, and I_3_ and I_4_ are the *S. aureus* counts in the control and film sample treatment groups, respectively.

(5) $$\mathrm{IR}\;\mathrm{of}\;E.\;\mathrm{coli}\;(\%)=\frac{I_{1}-I_{2}}{I_{1}}\times 100$$

(6) $$IR\;of\;S.\;aureus\;(\%)=\frac{I_{3}-I_{4}}{I_{3}}\times 100$$

#### 2.4.12. General Data-Processing Methods

All analyses were performed in triplicate, and the results were expressed as means ± standard deviations. The data were analyzed by one-way ANOVA, followed by Duncan’s multiple range test at a significance level of *p* < 0.05. Strawberry data were analyzed by one-way ANOVA, followed by Duncan’s multiple-range test, separately at each storage time; this approach does not test treatment × time interactions, which is acknowledged as a statistical limitation.

### 2.5. Application of TS/AX/PVA Films in Strawberry Preservation

#### 2.5.1. Sample Preparation

Fresh strawberry samples of uniform size and free of decay were randomly divided into four treatment groups, each comprising 60 strawberries (total 240 fruit): unsealed control, PE (sealed with commercial PE cling film), AX/PVA (sealed with TS-free AX/PVA film), and 6% TS/AX/PVA (sealed with 6% TS/AX/PVA film). Samples were withdrawn at six storage time points (days 0, 2, 4, 6, 8, and 10); at each time point, 10 fruit per treatment were destructively analyzed, distributed as 4 fruit assigned to decay-incidence assessment and 6 fruit assigned to the six remaining quality indices (weight loss, firmness, soluble solids content, titratable acidity, relative electrical conductivity, and vitamin C content). The complete allocation both of fruit per treatment and storage time is given in Table S6. Because the measurements are destructive, no single fruit was sampled repeatedly. Strawberry data were analyzed by one-way ANOVA, followed by Duncan’s multiple-range test, performed separately at each storage time; this approach does not model treatment × time interactions, which is acknowledged as a statistical limitation. The 6% TS/AX/PVA film was selected for the preservation trials as the working-optimum formulation (Section 3.1.4); the comparative preservation performance of the other formulations was not evaluated and remains a subject for future work.

#### 2.5.2. Calculation of Weight Loss Rate

Weight loss (WL, %) of strawberries during storage was calculated by Equation (7), where W_0_ is the initial weight (g) per group, and W_m_ is the weight (g) at storage time (m = 0, 2, 4, 6, 8, 10 d).

(7) $$\mathrm{WL}(\%)=\frac{W_{0}-W_{m}}{W_{0}}\times 100$$

#### 2.5.3. Decay-Incidence Rate

The decay status of fresh strawberries was evaluated visually. Strawberries with surface collapse, browning, or mold development were defined as decayed. The decay-incidence rate (DI, %) was determined by the counting method [20] and calculated using Equation (8), where F_0_ is the initial number of strawberries, and F_t_ is the number of decayed strawberries at storage time (t = 0, 2, 4, 6, 8, 10 d).

(8) $$DI(\%)=\frac{F_{t}}{F_{0}}\times 100$$

#### 2.5.4. Firmness

During the experiment, the outer 1 cm layer of each strawberry was removed. A handheld fruit hardness tester (GY-3, Dongguan Sanliang Measuring Instrument Co., Ltd., Dongguan, China) was used to apply vertical pressure to the flesh. The force reading was recorded. Measurements were performed in triplicate, and the mean value was taken as the final result.

#### 2.5.5. Relative Electrolytic Leakage

A 1.00 g strawberry pulp sample was immersed in 15.0 mL of ultrapure water and kept at room temperature for 1 h, and the initial conductivity (C_1_) was measured. After heating in a boiling water bath for 30 min and cooling to room temperature, the final conductivity (C_2_) was recorded. Relative conductivity was calculated using Equation (9), where C_1_ and C_2_ are the initial and final conductivities (μS/cm), respectively.

(9) $$Relative\;Electrolytic\;Leakage(\%)=\frac{C_{1}}{C_{2}}\times 100$$

#### 2.5.6. Titratable Acid

A 2.50 g strawberry pulp sample was homogenized, transferred to a 50.0 mL volumetric flask, and diluted to volume [21].

#### 2.5.7. Soluble Solid

A strawberry pulp sample (2.50 g) was ground in a mortar, and the supernatant was collected. Soluble solid content was measured with an Abbe refractometer in triplicate.

#### 2.5.8. Determination of VitC Content

The titer was determined by dye titration with standard VitC solution [22]. A 5.00 g sample was homogenized, diluted to 50.0 mL with 2.00% oxalic acid solution, and centrifuged. A 10.0 mL aliquot of the supernatant was titrated with 2,6-dichlorophenolindophenol solution, and the titration volume was recorded. All measurements were performed in triplicate, and the mean value was calculated.

#### 2.5.9. Statistical Considerations for Strawberry Data

Strawberry data were analyzed by one-way ANOVA, followed by Duncan’s multiple-range test, separately at each storage time; because this approach was applied independently to each time point, it does not test treatment × time interactions, which is acknowledged as a statistical limitation.

## 3. Results

### 3.1. Optimization of Formulations for Composite Films

#### 3.1.1. AX/PVA Ratio, Film Tensile Strength, and Elongation at Break

The effects of AX/PVA volume ratios on the thickness, tensile strength, and elongation at break of AX/PVA composite films are shown in Table S7. With increasing AX/PVA volume ratio, the tensile strength first increased and then decreased, while elongation at break declined continuously. As a rigid low-micron particulate material, AX was uniformly blended with PVA solution under ultrasonication. The enhanced hydrogen bonding improved tensile strength via a mechanism similar to short-fiber reinforcement. The tensile strength and elongation at break of the films at different AX/PVA volume ratios (1:9, 2:8, 3:7, 4:6, and 5:5) are reported as mean ± standard deviation in Table S7 (Supplementary Material). At a low loading (2:8), AX dispersed well and formed hydrogen-bonded networks with PVA, increasing tensile strength; above 2:8, excessive AX caused agglomeration and interfacial defects, reducing the mechanical performance. The 2:8 ratio was therefore selected as the working AX/PVA ratio.

#### 3.1.2. TS Loading, Film Tensile Strength, and Elongation at Break

Food-packaging materials require satisfactory mechanical properties to resist physical deformation during transport and storage, thus preserving packaged-food integrity. These properties determine the tensile strength and flexibility of packaging films. Given the poor film-forming ability of xylan, PVA acts as a structural scaffold to optimize its film-forming performance and reinforce the mechanical robustness of the composite film. Moreover, glycerol serves as an effective plasticizer to improve the flexibility of AX/PVA blend films [23,24]. Neat PVA films exhibit a tensile strength of 29.18 MPa and an elongation at break of 258.23%. As shown in Table 1, with increasing TS loading in AX/PVA blends, the tensile strength of composite films decreases gradually, while the elongation at break first increases and then decreases. This trend stems from the hydrophilicity of saponin and xylan, which elevates moisture uptake, leading to lower tensile strength but higher elongation. As a surfactant, TS weakens the interchain interactions and crystallinity of PVA macromolecules, thereby improving the elongation at break. In addition, as an efficient dispersant, TS alleviates AX agglomeration and enhances the interfacial compatibility between AX and PVA, which synergistically improves the mechanical properties of composite films.

#### 3.1.3. TS Loading, Film Thickness, and Moisture Characteristics

The thickness, moisture content, and water solubility of TS/AX/PVA films with different TS contents are summarized in Table 2. The data indicate that incorporation of either TS or AX alone increases film thickness. The neat AX/PVA film is 0.061 mm thick, while the 4% TS/PVA film is 0.064 mm. When both TS and AX are added to the PVA matrix, film thickness increases gradually with rising TS concentration. Thickness varies significantly among formulations, reaching a maximum of 0.085 mm at 8% TS loading. Among all tested films, the neat PVA film had a moisture content of 23.95%, which was mainly ascribed to the inherent strong hydrophilicity of PVA. A comparison between the AX/PVA composite film and 4% TS/PVA composite film showed that the addition of both AX and TS could effectively reduce the moisture content of the films. Notably, incorporating AX into the PVA matrix exhibited a more significant effect on lowering the moisture content of composite films. This is because AX contains abundant hydroxyl groups, which preferentially interact with the hydrophilic groups of PVA, reducing the binding sites for water molecules in the system and thereby decreasing the film’s moisture content. For the AX/PVA base film system, the moisture content of composite films first increased and then decreased with the increase in TS dosage, and the 6% TS/AX/PVA film achieved the lowest moisture content of 10.39%.

The decreased water solubility is mainly related to the intrinsic hydrophilicity of PVA, which possesses a higher hydroxyl group density than TS and AX. Table 2 shows the effect of TS dosage on the water solubility of AX/PVA composite films. The solubility declined significantly with the increase in TS content, which could be attributed to the hydrogen bonding between TS and the hydroxyl groups of PVA and AX, thereby reducing the number of hydrophilic sites [25]. This indicates that the incorporation of AX weakens the hydrophilicity of PVA, and TS endows the AX/PVA film system with improved water resistance. In this work, the water solubility of the prepared composite films (6%TS/AX/PVA) was slightly higher than that of urea-plasticized films (27.01%). The results confirm an obvious synergistic effect induced by the simultaneous addition of TS and AX [26].

#### 3.1.4. Determination of the Optimal Composite Film Formulation

The ternary film formulation was optimized via tuning AX/PVA ratio and TS loading. AX strengthens PVA at AX/PVA = 2:8 by hydrogen bonding, while excess AX agglomeration deteriorates mechanical properties. As surfactant and dispersant, TS improves ductility and AX dispersion yet cuts tensile strength at excessive loading. Film thickness rises with increased AX and TS. AX reduces moisture content via binding PVA hydrophilic sites; moisture content rises and then falls upon TS incorporation, optimized at 6 wt% TS. TS synergizes with AX to suppress solubility and boost water resistance. The optimal condition is AX/PVA = 2:8 and 6 wt% TS, yielding films with balanced mechanical and water-resistant performance.

In the absence of a formal desirability-function optimization, the 6 wt% TS was selected as a working optimum because it combined the lowest moisture content and water solubility with adequate tensile strength and the highest elongation at break; this selection reflects an empirical trade-off between mechanical robustness and water resistance rather than a statistically optimized composition.

### 3.2. Morphological Characteristics of Composite Films

#### 3.2.1. Appearance of TS/AX/PVA Films

A composite film was fabricated via solution casting, using AX/PVA as the film matrix and TS as the active component. The surface morphology and color of the films were documented photographically. Figure 1a(A–F) shows the appearance of TS/AX/PVA films with different TS loadings. With increasing TS concentration, the film color gradually darkened from colorless to yellowish brown, while excellent transparency was retained, as text underneath remained clearly visible. All five films exhibited smooth, uniform surfaces and consistent thickness, suggesting good compatibility between TS and the AX/PVA matrix.

#### 3.2.2. Electron Microscopic Analysis of Xylan Extracted from *Camellia oleifera* Husks

The morphology of AX was characterized by transmission electron microscopy (TEM) and scanning electron microscopy (SEM), and its particle size distribution was further analyzed. Figure 1b(A,B) are TEM images of xylan with scale bars of 0.5 μm and 0.2 μm, and Figure 1b(C) is the corresponding SEM image with a 1.0 μm scale bar. As shown in Figure 1b, xylan particles display irregular shapes and assemble into a porous network structure. The particles readily aggregate due to their high surface energy. The particle size distribution was quantified based on SEM images. As presented in Figure S20, 80% of xylan particles are sized between 0.5 and 2 μm; the fraction of particles in the 1–1.5 μm range reaches the maximum of 29.42%. These submicron/micron-scale xylan particles can form tortuous diffusion pathways within the blended films, which effectively enhance the barrier properties against water-vapor and gas permeation. Figure 1a shows representative photographs of the films, illustrating their general appearance. These images are intended for illustration only and should not be interpreted as quantitative color or transmittance measurements; light transmittance was measured instrumentally by UV-Vis spectroscopy.

### 3.3. Chemical Composition of Composite Films

Because the purified tea saponin had a purity of 71.28%, a fraction of co-extracted components may contribute to the observed properties; accordingly, the reported antioxidant, antimicrobial, and functional activities are attributed to the purified extract as a whole rather than exclusively to tea saponin itself.

#### 3.3.1. FT-IR Analysis of TS Extracted from *Camellia oleifera* Husks

The functional groups of the tea-saponin aglycone were characterized using FT-IR spectroscopy, as shown in Figure S9. A broad absorption band at 3425 cm^−1^ corresponds to the characteristic stretching vibration of hydroxyl groups. The absorption peaks at 2935, 1452, and 1339 cm^−1^ are attributed to the stretching and bending vibrations of methyl and methylene groups. The peak at 1719 cm^−1^ is assigned to the stretching vibration of aldehyde groups, while the signal at 1613 cm^−1^ arises from the stretching vibration of C=C bonds. The absorption at 1051 cm^−1^ is attributed to the bending vibration of C–O–H. These characteristic peaks are consistent with those reported in previous studies for TS obtained via ultrasonic alcohol extraction and ultrasonic water-bath extraction, and they closely match the key spectral features of a TS standard. These results confirm that the extracted substance is indeed TS and indicate that the decolorization process did not alter its molecular structure.

#### 3.3.2. Color Reaction Analysis of TS Extracted from *Camellia oleifera* Husks

A positive Molish reaction was found for TS extracted from *Camellia oleifera* husks. A characteristic purple zone formed at the bottom of the test tube, and the results were consistent with expectations, verifying the isolated compound as TS (Figure S10).

#### 3.3.3. FT-IR Analysis of Xylan Extracted from *Camellia oleifera* Husks

As shown in Figure S19, the FT-IR spectrum of AX exhibited a broad peak at 3295 cm^−1^, which corresponds to the stretching vibration of hydroxyl groups. The xylan hemicellulose structure contains a large number of hydroxyl groups, contributing to its hydrophilic nature. The absorption peak observed at 2920 cm^−1^ is attributed to the stretching vibration of C–H functional groups. The peak at 1066 cm^−1^ serves as an important marker of the intrinsic characteristics of xylose molecules, representing the stretching vibration of the ether bond (C–O–C). The absorption peak at 902 cm^−1^ is specifically assigned to the unique configuration of *β*-D-xylose, indicating that the sugar units are linked via *β*-glycosidic bonds. The region spanning 1170–1000 cm^−1^ contains characteristic peaks of the arabinoxylan backbone, confirming that the primary component of the extracted sample is xylan. The absorption peak at 1162 cm^−1^ indicates the presence of arabinose side groups. Based on the infrared spectral analysis, it can be concluded that the xylan extracted from *Camellia oleifera* husks is composed predominantly of xylose. These characteristic peaks are consistent with the infrared features reported for corn-cob xylan and for xylan extracted via ultrasonic-assisted low-temperature alkaline extraction.

#### 3.3.4. Nuclear Magnetic Resonance Analysis of Xylan Extracted from *Camellia oleifera* Husks

To elucidate the chemical structure characteristics of AX, this study employed NMR spectroscopy for precise molecular structure characterization, with particular focus on the main-chain configuration and side-chain structure. Figures S21 and S22 present the ^1^H NMR and ^13^C NMR spectra of the AX, respectively. Signal assignments were made based on relevant literature and comparative analysis with reported data.

As shown in Figure S21, the ^1^H NMR spectrum of the xylan sample reveals characteristic anomeric proton signals. Typically, anomeric protons of *α*-configured sugars appear in the *δ*_H_ 5.1–5.8 ppm range, while those of *β*-configured sugars resonate between *δ*_H_ 4.4 and 4.8 ppm. The peaks at *δ*_H_ = 4.36, 3.53, 3.46, 3.35, 3.33, and 4.08 ppm were assigned to the H-1, H-2, H-3, H-4, H-5a, and H-5b absorption signals of the *β*-D-xylopyranose unit, respectively. A minor peak at *δ*_H_ = 1.20 ppm was attributed to the H-6 absorption of rhamnose residues, indicating the presence of minor side-chain components. The strong signal at *δ*_H_ = 4.73 ppm was assigned to residual deuterium oxide (D_2_O) solvent.

In the ^13^C-NMR spectrum (Figure S22), anomeric carbons of polysaccharides mainly resonate at *δ*_C_ 92–110 ppm. Characteristic carbon signals from sugar residues of polysaccharides are observed in the range of *δ*_C_ 55–85 ppm. Among these, the signals at *δ*_C_ 58–68 ppm are assigned to exocyclic methylene (–CH_2_–) carbons, i.e., C6 of pyranose rings and C5 of furanose rings. The multiple signals within *δ*_C_ 68–85 ppm correspond to endocyclic C2–C4 carbons of sugar residues other than anomeric carbons. Severe signal overlap is detected in this region, which is a typical feature of the ^13^C NMR spectra of polysaccharides. Specifically, the chemical shifts at *δ*_C_ = 105.9, 78.3, 75.1, 76.5, and 65.8 ppm were assigned to the C-1, C-2, C-3, C-4, and C-5 positions of the arabinose side chains attached to the *β*-D-xylose backbone, respectively.

### 3.4. Physicochemical Properties of Composite Films

#### 3.4.1. Light Transmittance Properties of TS/PVA/AX Films

Light transmittance of packaging films directly influences food-preservation efficiency. High transmittance triggers photo-oxidation, accelerating nutrient loss and spoilage, including lipid oxidation and enzymatic browning, in fruits and vegetables. In contrast, low transmittance suppresses sprouting and discoloration of light-sensitive components, extending shelf life [27]. Since transmittance correlates with UV barrier properties and application conditions, a rational balance between visual inspection and photoprotection is required. Thus, transmittance serves as a key parameter for evaluating packaging film’s applicability and preservation performance [28]. Neat PVA exhibits the highest transmittance (Figure 2a). Incorporation of AX and TS into the PVA matrix markedly reduces film transmittance (Figure 2b), due to intermolecular hydrogen bonds that induce molecular rearrangement and aggregation, forming ordered structures and lowering transparency [29]. Notably, within 350–450 nm, the transmittance of TS/AX/PVA films gradually decreases with increasing TS loading. Abundant hydroxyl and carboxyl groups in TS form hydrogen bonds with the AX/PVA matrix, raising film density and blocking UV transmission pathways [30].

#### 3.4.2. Thermal Stability of TS/AX/PVA Films

This study systematically characterized the thermal stability of blended films using thermogravimetric (TG) and derivative thermogravimetric (DTG) analyses. As presented in Figure 2c,d, all six blended films exhibited similar three-stage degradation profiles, in contrast to the neat PVA film. The first degradation stage (50–150 °C, 0–5% mass loss) was mainly attributed to the evaporation of adsorbed moisture and the decomposition of low-molecular-weight additives. PVA exhibited a more significant weight loss in this stage owing to its stronger moisture adsorption capacity. The second stage (150–250 °C, 23–50% mass loss) corresponded to the partial degradation of glycerol, AX, PVA, and the triterpenoid saponin structure in TS. The third stage (250–475 °C) involved the complete cleavage of polymer backbones and the formation of carbonaceous residues or inorganic ash, including the scission of PVA C–C backbones, breakdown of AX chains, and carbonization of TS residues [31]. At 600 °C, the residual mass percentages of the six composite films were 5.64%, 14.31%, 6.51%, 14.28%, 13.45%, and 12.57%, respectively. Overall, the TS/AX/PVA films demonstrated superior thermal stability relative to neat PVA, confirming that the synergistic incorporation of TS and AX effectively enhances the thermal stability of the composite films.

#### 3.4.3. Infrared Spectra of TS/AX/PVA Films

The FT-IR spectra in Figure 2e reflect hydroxyl peak intensity changes caused by hydrogen bonding among glycerol, TS, PVA and AX. The band at 3000–3600 cm^−1^ corresponds to O–H stretching, and its declining intensity with increasing TS content suggests fewer hydrophilic hydroxyl groups. Hydrogen-bond formation further offers a partial rationale for the compatibility between PVA and AX. Meanwhile, the spectral evidence indicates enhanced hydrophobicity of the AX/PVA composite films upon the introduction of glycerol and TS. Furthermore, the detection of an asymmetric alkyl C–H stretching peak at 2940 cm^−1^ confirms the satisfactory incorporation of glycerol within the film-forming matrix. Compared with the FT-IR spectrum of neat PVA, the 4%, 6%, and 8% TS/AX/PVA films display a novel absorption peak at 1711 cm^−1^ ascribed to C=O stretching vibrations, suggesting the formation of ester bonds. The 4%, 6%, and 8% TS/AX/PVA films display a novel absorption peak at 1711 cm^−1^ ascribed to C=O stretching vibrations. Because tea saponin itself shows carbonyl absorption near 1719 cm^−1^, this band alone does not confirm the formation of new ester bonds; the proposed esterification/hydrogen-bonding interactions should be regarded as a plausible interpretation requiring confirmation by complementary techniques such as solid-state ^13^C NMR or XPS.

#### 3.4.4. Water-Vapor Permeability of TS/AX/PVA Films

The water-vapor permeability (WVP) of food-packaging films not only governs their moisture exchange capacity with the external environment; it also exerts a critical influence on the physical properties of packaged food as well as the risk of microbial proliferation during storage. Figure 2f presents the effect of TS loading on the WVP of AX/PVA films. The 6% TS/AX/PVA film exhibits the minimum water-vapor permeability (WVP = 2.66 × 10^−10^ g·m·m^−2^·s^−1^·Pa^−1^) among the tested films, exhibiting the best water vapor-barrier performance. Appropriate TS loading tailors the porous structure of the polymer matrix, reduces water-diffusion channels and enhances the water-vapor barrier; excessive TS loading disrupts the hydrogen-bonding architecture and raises the WVP. Hydrogen bonds between AX and PVA produce a compact microstructure resisting water permeation. Meanwhile, AX forms a dense fibrous network and seals surface pores to restrict air penetration [32]. Meanwhile, appropriate TS loading tailors the porous structure of the polymer matrix, reduces water diffusion channels, and enhances water vapor-barrier performance. Nevertheless, excessive TS loading disrupts the intrinsic hydrogen-bonding architecture of PVA, increasing unbound hydroxyl moieties along polymer chains and raising the WVP of the composite film.

### 3.5. Biological Activity of Composite Films

#### 3.5.1. Antioxidant Effects of TS

DPPH radical scavenging assay was employed to assess the antioxidant capacity of purified TS extract, following a modified protocol from existing research on plant-derived saponins [33]. Figure 3a shows that the DPPH scavenging rate increased gradually with TS concentration ranging from 0.25 to 3.0 mg/mL, before leveling off above 2.0 mg/mL. At all tested concentrations, the extract exhibited weaker scavenging activity than the VitC control. Nevertheless, a maximum scavenging rate of 81.87% was achieved at 3.0 mg/mL, confirming the strong antioxidant potential of TS.

#### 3.5.2. Antibacterial Activity of TS

Figure 3b,c present the antibacterial performance of TS. The inhibition-zone diameters of the two test bacteria increased remarkably with elevated TS concentration, confirming a positive dose–response relationship. TS showed limited inhibitory activity against both *E. coli* and *S. aureus* at concentrations lower than 100 mg/mL. Meanwhile, *S. aureus* was always more vulnerable to TS, reflecting distinct antibacterial selectivity between the two species. At 200 mg/mL and 300 mg/mL, the inhibition zones for *S. aureus* were substantially wider than those for *E. coli*. Such discrepancies stem from differences in cell-envelope architecture. As a Gram-negative bacterium, *E. coli* features a double membrane containing lipopolysaccharides in the outer layer, whereas Gram-positive *S. aureus* has merely a single cytoplasmic membrane. This structural distinction renders *E. coli* more resistant to TS, in line with prior research findings. Previous work has also clarified the antibacterial mechanisms of saponins. Zhao et al. demonstrated that saponins extracted from tea leaves exert potent activity against *E. coli* and *S. aureus* by altering membrane permeability, disrupting membrane integrity, and inhibiting bacterial growth [34]. The apparent reversal in susceptibility between free tea saponin and the tea saponin-containing films does not reflect a change in intrinsic bacterial sensitivity; rather, it arises from differences in TS availability and exposure mode—a freely soluble compound in the former assay versus TS entrapped within the film matrix in the latter.

#### 3.5.3. Antibacterial Properties of TS/AX/PVA Films

The antibacterial activity of the composite films was assessed via an immersion co-culture assay. TS/AX/PVA films with varied TS loadings were incubated in uniform *E. coli* and *S. aureus* suspensions for 90 min, and viable cell counts were quantified by the spread-plate technique. As shown in Figure 3d, dense bacterial growth was observed on plates treated with neat PVA or AX/PVA films, while colony counts of *E. coli* and *S. aureus* were drastically reduced by TS-loaded composite films. The antibacterial activity increased with TS concentration, with *E. coli* being more sensitive than *S. aureus.* The 8% TS/AX/PVA film exhibited optimal performance (sparse colonies only; 95% antibacterial efficiency). TS acts by disrupting the integrity of bacterial cell walls and membranes, impairing peptidoglycan cross-linking and the phospholipid bilayer to induce osmotic imbalance. It also inhibits bacterial motility and biofilm formation, suppressing growth and reducing virulence [35]. Because the immersion co-culture assay cannot distinguish between contact-dependent activity and activity arising from the release of soluble tea saponin, nor between bacteriostatic and bactericidal effects, we refer to overall growth inhibition rather than to a specific mechanism. Photographs of plates alone are insufficient and have certain limitations.

#### 3.5.4. The Antioxidant Performance of TS/AX/PVA Films

The antioxidant activity of TS/AX/PVA films was evaluated via the DPPH radical scavenging assay. As shown in Figure 3e, neat PVA and AX/PVA composite films exhibited low DPPH scavenging rates. As a film-forming matrix, PVA lacks potent reducing groups (e.g., phenolic hydroxyls and thiols) and thus shows negligible intrinsic antioxidant activity. Although alkaline treatment slightly improves the antioxidant activity of xylan [36], its antioxidant capacity still remains limited. Notably, the DPPH-scavenging activity of the composite films increased significantly with increasing TS content, confirming a clear positive correlation between TS concentration and the antioxidant performance of the composite films. Antioxidant capacity was evaluated with a single DPPH assay; because DPPH results may be influenced by film coloration/turbidity and do not capture all radical-scavenging pathways, the measured activity should be regarded as preliminary.

### 3.6. Degradability of TS/AX/PVA Films

Soil-burial testing was performed to evaluate the biodegradability of the composite films. As illustrated in Figure 4A–F, the as-prepared films were monitored over a 30-day period. During the initial 5 days, all samples remained morphologically intact but became pliable due to moisture absorption from the soil, with groups a, b, and c showing the most pronounced changes. Structural deformation and disintegration then occurred between days 5 and 15, and nearly complete degradation was observed for all films by day 30. Relative to neat PVA, films containing TS or AX alone, as well as AX/TS composites, exhibited more significant structural changes throughout degradation. The results confirmed that TS/AX/PVA films near-complete visual disintegration within 30 days of soil burial with appropriate temperature, humidity, and abundant microbial communities, and because no mass-loss or mineralization kinetics were measured, this observation is best described as soil-burial (visual) degradation rather than complete biodegradation. Biodegradation of PVA depends on specific microbial consortia [37], whereas AX is readily hydrolyzed by microbial xylanases into sugars that act as carbon sources, promoting microbial colonization and matrix erosion. Accordingly, AX/PVA films are more susceptible to microbial attack and display accelerated degradation kinetics. Although TS exhibits mild antimicrobial activity, its hydrophilicity favors gradual decomposition and indirectly enhances the overall biodegradation process [38].

### 3.7. Fresh-Keeping Performance of TS/AX/PVA Films for Strawberries

Strawberries have fragile epidermal tissues without a protective outer rind, making them highly susceptible to multiple stresses during post-harvest circulation (transport, storage, and marketing), including physical damage, respiratory loss, and microbial infection [39,40]. Flavor and water loss occur within 3 days post-harvest, sharply reducing commercial quality. Thus, developing protective packaging materials is critical to delaying strawberry-quality deterioration. Using TS from *Camellia oleifera* husks as the main active ingredient, we prepared TS/AX/PVA films with excellent barrier, mechanical, antibacterial, and antioxidant properties in Section 3.1, Section 3.2, Section 3.3, Section 3.4 and Section 3.5. This section evaluates their preservation efficacy for fresh strawberries compared with commercial cling film. Key indicators were monitored during storage: weight loss rate, decay rate, firmness, relative electrical conductivity, titratable acidity, soluble solids content, and VitC content [41]. These results provide a reference and technical support for maintaining strawberry quality and extending shelf life.

#### 3.7.1. Weight Loss Rate Analysis

Strawberries with high moisture content are prone to transpirational water loss during storage, causing weight loss and shriveling that greatly reduce their commercial value. As a key indicator, weight loss rate directly reflects freshness dynamics, and Figure 5a shows its time-dependent variation among groups. The weight loss rate of all groups increased with storage time due to aerobic respiration and water evaporation [42]. After 10 days, the unsealed control group reached 29.14%, the 6% TS/AX/PVA film group was 17.52%, and the PE group exhibited the lowest value of 3.95%. The AX/PVA and 6% TS/AX/PVA film groups showed intermediate weight loss rates. This can be explained by two factors: First, PVA has excellent water barrier properties, and intermolecular interactions among PVA, AX, and TS further enhance the water vapor-barrier effect, reducing weight loss in strawberries. The lower weight loss of the film-packaged groups is attributed to the improved water vapor-barrier properties of the composite films, which reduced transpirational water loss during storage [43].

#### 3.7.2. Changes in Appearance and Decay Rate

Decay rate is a critical, intuitive indicator for evaluating fruit-preservation efficacy. Figure 6 shows morphological changes in fresh strawberries in each group over 10 days of storage, with color changes, tissue softening, and mold growth clearly annotated. The unsealed control group first showed pericarp darkening on day 2. By day 4, all samples exhibited color deepening, and localized softening appeared in the unsealed control group. On day 6, all groups except the 6% TS/AX/PVA film group developed decay lesions; the unsealed control and PE groups had 2–3 decay spots with obvious white mold colonies, losing commercial value entirely. At 8–10 days, spoilage in the unsealed control and PE groups worsened sharply with visible mycelial growth, suggesting severe microbial decay, and mycelial proliferation was denser in the PE group. This stems from the fragile strawberry epidermis causing rapid postharvest water loss, while direct flesh-air contact accelerates oxidation, favoring mold growth and resulting in oxidative browning and mold development [44]. After 10 days of storage, the 6% TS/AX/PVA group maintained the best freshness, with only moderate softening and slight spotting at the calyx end. The 6% TS/AX/PVA group maintained the lowest decay incidence throughout storage; this reduction in decay is reported as an empirical preservation outcome of the composite packaging. Because no antifungal or direct strawberry-microbiological assessment was performed, the underlying cause cannot be attributed to antimicrobial or antifungal activity and requires further investigation.

Figure 5b shows the temporal decay-rate variations among strawberry groups. The unsealed control and PE groups presented significantly higher decay incidence than the 6% TS/AX/PVA group. The AX/PVA composite film effectively reduced water loss and mechanical damage, thus lowering the risk of pathogenic fungal infection. TS incorporation enhanced the water-vapor barrier of AX/PVA film and formed a multifunctional antioxidant and antibacterial barrier, further retarding strawberry spoilage. Mold lesions appeared in the PE group on day 6, whereas only slight mold spots were observed at the calyx end of the 6% TS/AX/PVA group on day 8. The TS/AX/PVA film effectively blocked air intrusion and inhibited oxidation and microbial growth. Due to the poor water and gas permeability of PE film [45], the strawberry decay rate reached 96% by day 10, indicating its limited long-term preservation effect.

#### 3.7.3. Hardness

Hardness is a quantitative indicator for evaluating strawberry texture changes during storage and is directly correlated with freshness [46]. Figure 5c shows the effects of different composite films on fresh-strawberry hardness. Over 10 days of storage, hardness decreased continuously in all groups due to natural water evaporation and accelerated degradation of cell wall components such as pectin and cellulose, causing tissue softening and hardness loss [47]. Both AX/PVA and 6% TS/AX/PVA groups outperformed the unsealed control group in retarding fruit softening. Notably, strawberries packaged with the 6% TS-modified AX/PVA film exhibited the slowest hardness decline and the highest final hardness among all groups. This is due to the intrinsic antibacterial activity of TS, which reduces softening and decay, improving hardness retention. The unsealed control group had a much higher water evaporation rate; prolonged storage and worsening spoilage accelerated cell wall degradation, leading to severe softening. In contrast, the 6% TS/AX/PVA group showed minimal hardness variation and better hardness, attributed to the excellent water- and oxygen-barrier properties of the composite film that suppressed postharvest respiration and delayed water transpiration [48].

#### 3.7.4. Relative Conductivity

Relative conductivity is a key physiological and biochemical parameter reflecting plant cell membrane integrity, and directly indicates changes in cell membrane permeability under stress. It is widely used as a reliable index to evaluate stress tolerance and quantify cellular damage in fruits and vegetables [49]. Stress conditions such as adverse environments and mechanical damage reduce cell membrane stability and impair membrane proteins. This damage causes leakage of cytoplasmic fluid, leading to a significant rise in relative conductivity [50].

Figure 5d shows the effects of different composite films on the relative conductivity of fresh strawberries. Relative conductivity increased continuously in all groups during storage, with all treatment groups maintaining significantly lower values than the unsealed control group. On day 2, relative conductivity was similar across all groups. From day 2 to day 10, the unsealed control and PE groups showed a rapid increase, reaching 75.65% and 68.25% on day 10, respectively, indicating progressive cell membrane damage during strawberry spoilage. In contrast, the 6% TS/AX/PVA group maintained a low relative conductivity, which was 18.43% lower than that of the unsealed control group on day 10. These results demonstrate that the 6% TS/AX/PVA film retards senescence and deterioration, alleviates cell membrane damage, and preserves cellular integrity, consistent with the visual quality of strawberries.

#### 3.7.5. Titratable Acidity

During postharvest storage, strawberries undergo continuous ripening and senescence. Sugars accumulate and degrade simultaneously, while organic acids are converted to other metabolites to sustain cellular metabolism, and their contents directly determine the sensory quality and storage stability of strawberries [51]. Figure 5e shows the effects of different composite films on the titratable acidity (TA) of fresh strawberries. TA decreased continuously in all groups, and the unsealed control group exhibited the fastest reduction, falling to 0.32% on day 10, with a 0.53% drop from the initial value. On day 2, the 6% TS/AX/PVA group maintained the highest TA level and the strongest inhibition of TA loss, thanks to its excellent CO_2_ barrier property that suppressed respiratory metabolism and mitigated TA degradation in the early storage stage. Throughout storage, this group showed minimal TA loss and retained 0.51% TA on day 10. These results confirm that the 6% TS/AX/PVA film effectively reduces oxygen permeability, lowers respiratory metabolism, slows the conversion and consumption of organic acids, and thus preserves strawberry quality during storage.

#### 3.7.6. Soluble Solids Content

Soluble solids content (SSC), a key physiological indicator comprising sugars, organic acids, and minerals, reflects fruit development and internal quality [52]. Figure 5f presents the dynamic changes in strawberry SSC during storage. In all samples, strawberry SSC exhibited an initial increase, followed by a gradual decrease. Specifically, SSC rose from day 0 to day 4 due to the hydrolysis of macromolecular carbohydrates into soluble sugars [53], but it declined from day 2 to day 10, as prolonged storage promoted continuous respiration and soluble sugar consumption. The PE-film group showed the sharpest SSC reduction, which was attributed to its strong water- and gas-barrier properties. In the early storage stage, aerobic respiration rapidly depleted oxygen, whereas anaerobic metabolism was activated in the later period, consequently accelerating sugar consumption, disrupting cell wall osmotic balance, and exacerbating fruit-quality deterioration.

#### 3.7.7. Analysis of VitC Content Variation

Changes in VitC retention in fresh strawberries treated with different composite films are presented in Figure 5g. VitC content decreased continuously in all groups during storage, mainly due to exogenous oxidation, endogenous respiratory metabolism, and microbial contamination [54]. The PE group exhibited the most severe VitC loss and fastest degradation rate, which differed significantly from the other three groups (*p* < 0.05); furthermore, only 15.56% of initial VitC remained on day 10. This was caused by the high gas barrier of PE film, which reduced internal oxygen and, in turn, negatively affected VitC retention [55]. In contrast, the 6% TS/AX/PVA group showed the slowest VitC degradation, retaining 26.64 mg/100 g VitC on day 10, representing a 71.21% increase compared with that of the PE group. The composite film inhibited respiration and exerted moderate antioxidant activity, thereby effectively reducing VitC oxidation [56]. Overall, the 6% TS/AX/PVA film most effectively alleviates VitC degradation and maintains the nutritional quality of strawberries.

#### 3.7.8. Preservation Mechanisms of Fresh Strawberries

Figure 7 depicts the dynamic interaction between the TS/AX/PVA film and strawberry surfaces, demonstrating a self-regulated vapor-phase antimicrobial process. The following processes are plausible hypotheses inferred from the film microstructure and measured water vapor-barrier properties and were not directly measured in this study: (i) tea-saponin release/migration toward the fruit, (ii) water vapor-mediated transport, (iii) O_2_ depletion, and (iv) CO_2_ accumulation in the headspace. These hypotheses remain to be verified by direct measurements of TS migration and headspace gas composition. With high moisture content and delicate epidermal tissues, strawberries constantly lose water via transpiration after harvest, generating gaseous H_2_O in the headspace. Serving as an effective physical barrier against exogenous microorganisms, oxygen and other contaminants in the enclosed system, the TS/AX/PVA film also possesses a microporous structure that enables reversible water-vapor adsorption and desorption. The film first adsorbs ambient moisture, followed by desorption upon reaching saturation. The condensed moisture dissolves and mobilizes TS embedded within the hydrophilic AX/PVA network. Released TS molecules are carried by desorbing water vapor and migrate toward strawberries under Brownian motion. Upon contact, TS-laden microdroplets inhibit spoilage organisms, including molds, by interfering with microbial cell wall synthesis, achieving potent antimicrobial effects without direct liquid contact. Relative to the unsealed control (CK), the 6% TS/AX/PVA film reduced strawberry weight loss and decay during storage; its day-10 weight loss (17.52%) was higher than that of the commercial PE film (3.95%), because the composite film is more permeable to water vapor than the dense PE film, but it delayed spoilage and maintained fruit quality more effectively than PE. Additionally, the semi-closed atmosphere elevates CO_2_ concentration and restricts O_2_ penetration. Together with active TS, this dual effect synergistically retards oxidative browning and fungal proliferation. The processes described here—moisture adsorption/desorption, dissolution and transport of tea saponin toward the fruit, and reduced O_2_ and elevated CO_2_ in the headspace—are plausible hypotheses inferred from the film microstructure and properties; headspace gas composition and tea-saponin release/migration were not directly measured and therefore remain to be verified. Because only water-vapor transport was experimentally measured, the films are described as exhibiting improved water vapor-barrier performance; no claim of oxygen or CO_2_-barrier properties is made, and any related statements are presented as inferences requiring direct measurement.

### 3.8. Limitations and Future Work

The microbial panel (*E. coli* DH5α and *S. aureus*) served only as a preliminary screening of film antibacterial activity; its direct relevance to fungal strawberry spoilage is limited, no antifungal organism was tested, and any suppression of strawberry mold cannot be inferred from the present data. These limitations define clear directions for future research, including antifungal testing against *Botrytis cinerea*, direct measurement of TS migration, headspace gas composition, and quantitative biodegradation.

## 4. Conclusions

In this work, TS and AX were sequentially extracted from abundant postharvest *Camellia oleifera* husk wastes, and novel biodegradable TS/AX/PVA films were successfully fabricated via solution casting. The incorporation of AX effectively optimized the mechanical strength and degradability of the PVA matrix, while the introduction of biomass-derived TS endowed the composite films with favorable antioxidant and antibacterial capacities. Compared with neat PVA and traditional polymer films, the optimized composites exhibited enhanced hydrophobicity, demonstrated superior barrier performance, and underwent near-complete visual disintegration within 30 days of soil burial; because no mass-loss or mineralization kinetics were measured, this is described as visual (soil-burial) disintegration rather than complete biodegradation. In practical preservation tests, the composite films reduced postharvest strawberry weight loss and decay (empirical outcome); the proposed mechanism involving tea-saponin migration and modified-atmosphere effects is presented as a hypothesis requiring direct verification. This study realizes the high-value valorization of agricultural by-products. Their potential for strawberry preservation is preliminary and would require migration/safety and sensory evaluation, while tea-saponin migration and modified-atmosphere effects remain hypotheses for future study. The fabricated eco-friendly composite films can serve as promising alternatives to non-degradable plastic packaging materials. This work provides a feasible strategy for the fabrication of low-cost, multifunctional, and sustainable bio-based packaging films, showing broad application potential in postharvest fresh-fruit preservation.

## Figures and Tables

**Figure 1:**
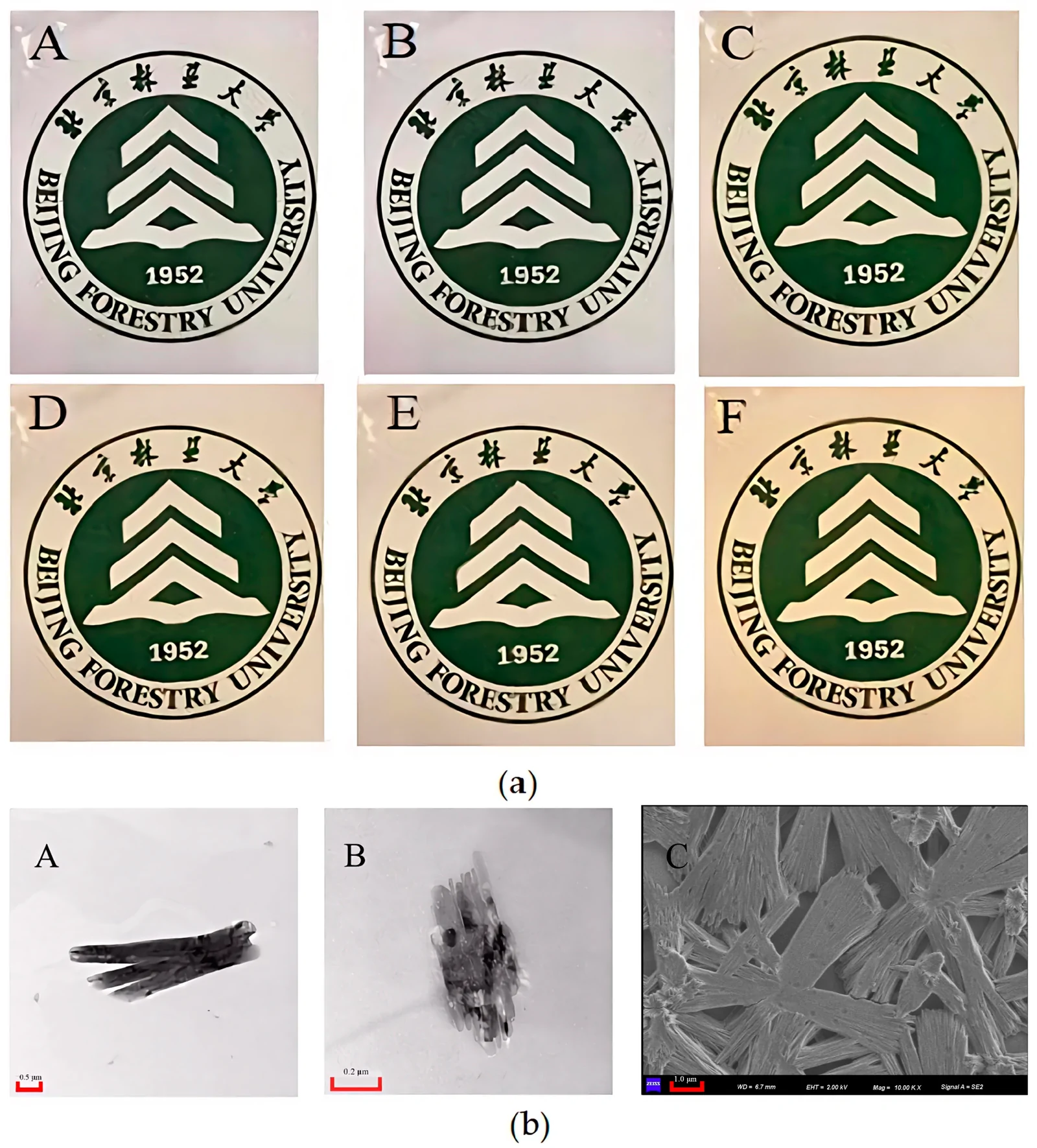
(**a**) Representative photographs illustrating the general appearance of the composite films ((**A**) PVA, (**B**) AX/PVA, (**C**) 4% TS/PVA, (**D**) 4% TS/AX/PVA, (**E**) 6% TS/AX/PVA, and (**F**) 8% TS/AX/PVA); shown for illustration only. (**b**) TEM and SEM micrographs of xylan ((**A**) 0.5 μm TEM, (**B**) 0.2 μm TEM, and (**C**) 1.0 μm SEM).

**Figure 2:**
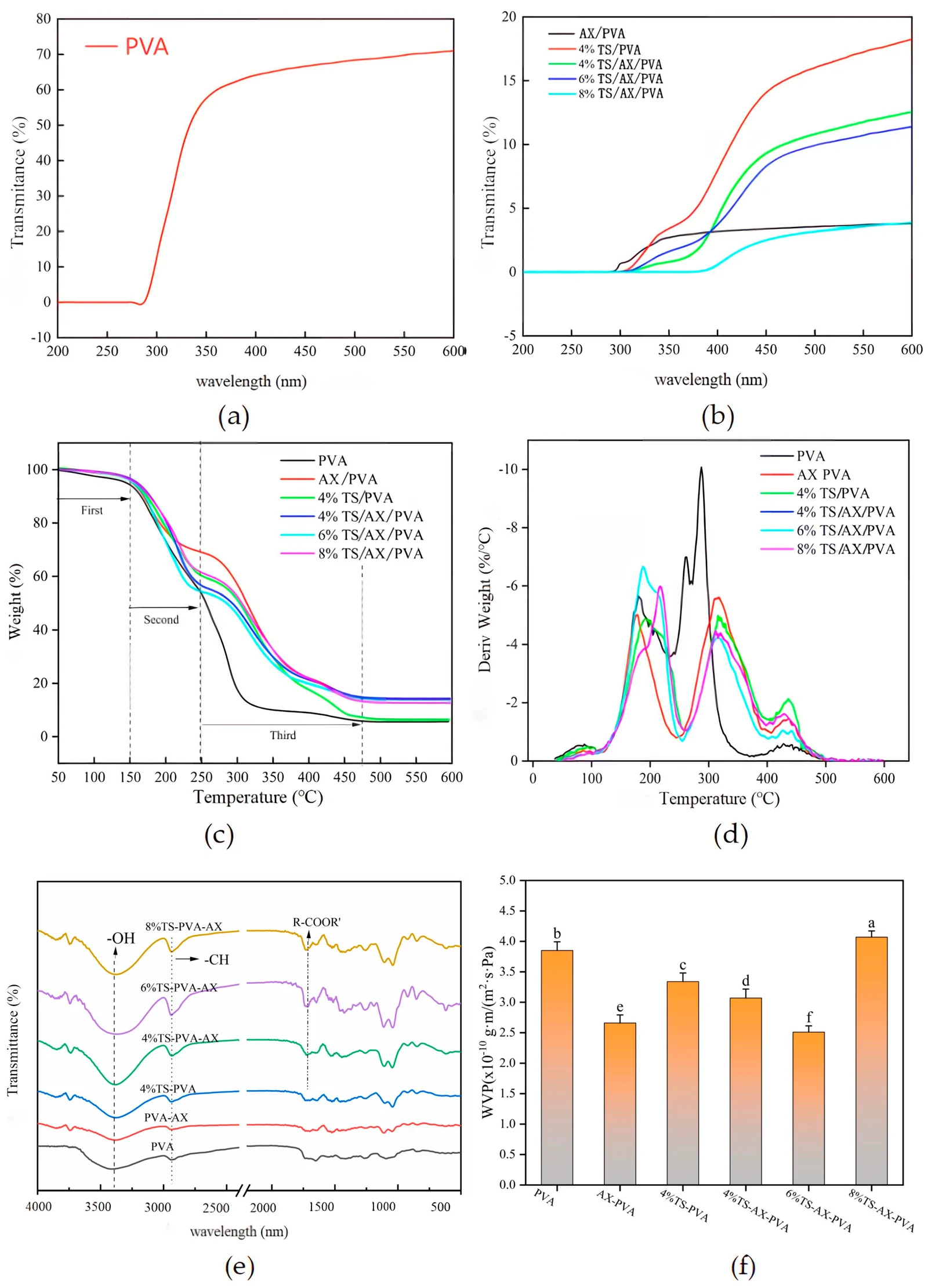
(**a**) UV barrier of neat PVA Film. (**b**) UV barrier of TS/AX/PVA films loaded different amounts of TS. (**c**) TG curves. (**d**) DTG curves. (**e**) Infrared spectra of different composite films. (**f**) Water-vapor permeability (WVP) of different composite films. Values followed by different lowercase letters are significantly different at *p* < 0.05 according to Duncan’s multiple-range test.

**Figure 3:**
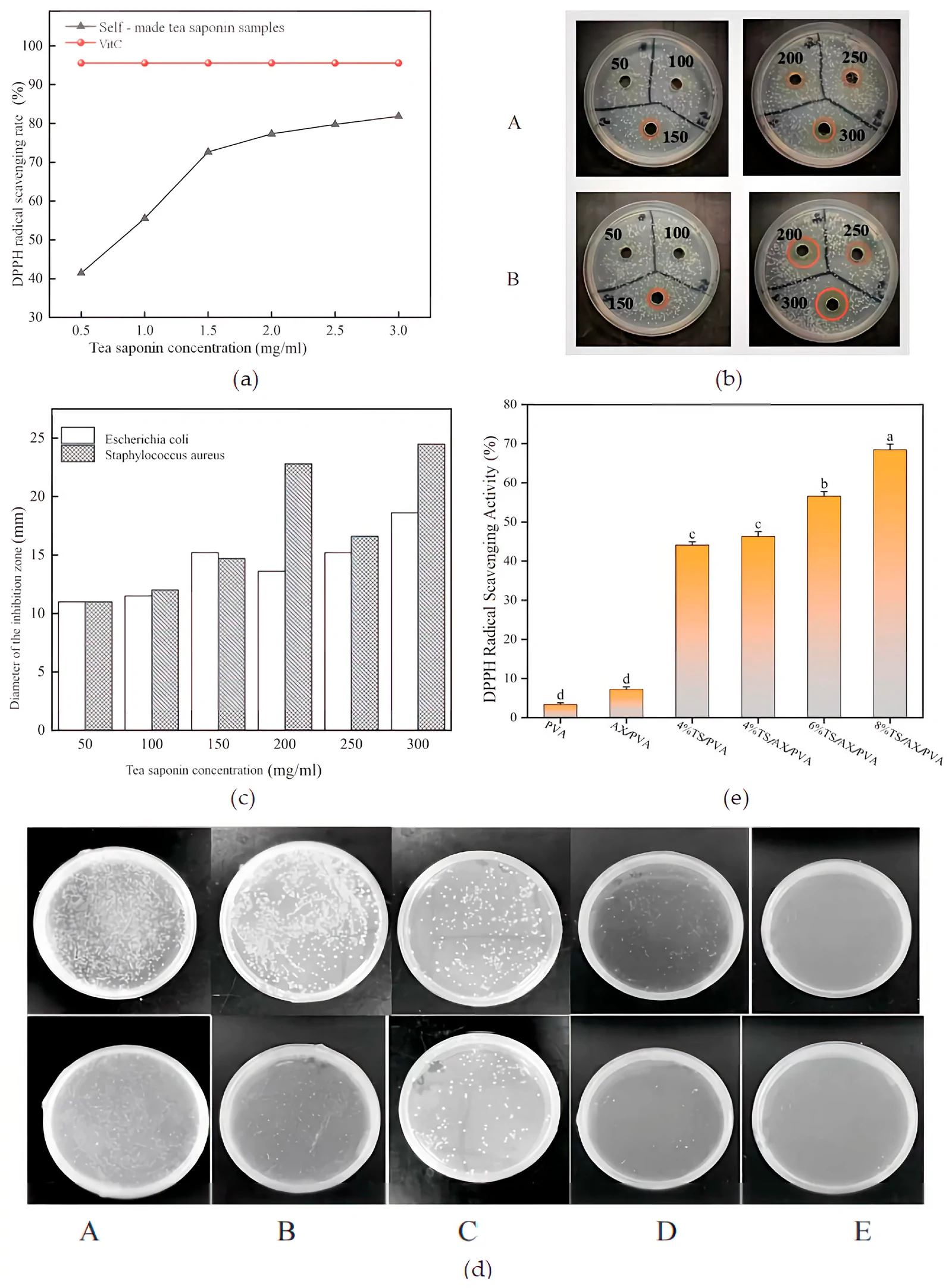
(**a**) Antioxidant effects of TS at different concentrations. (**b**) Antibacterial activity of TS at different concentrations against *E. coli* and *S. aureus*: (**A**) *E. coli* and (**B**) *S. aureus*. (**c**) Antibacterial inhibition zones of different TS concentrations against *E. coli* and *S. aureus*. (**d**) Antibacterial activity of different composite films against *E. coli* and *S. aureus*: (**A**) neat PVA film; (**B**) AX/PVA base film; and (**C**–**E**), 4%, 6%, and 8% TS/AX/PVA films. (**e**) Antioxidant properties of different films. Values followed by different lowercase letters are significantly different at *p* < 0.05 according to Duncan’s multiple-range test.

**Figure 4:**
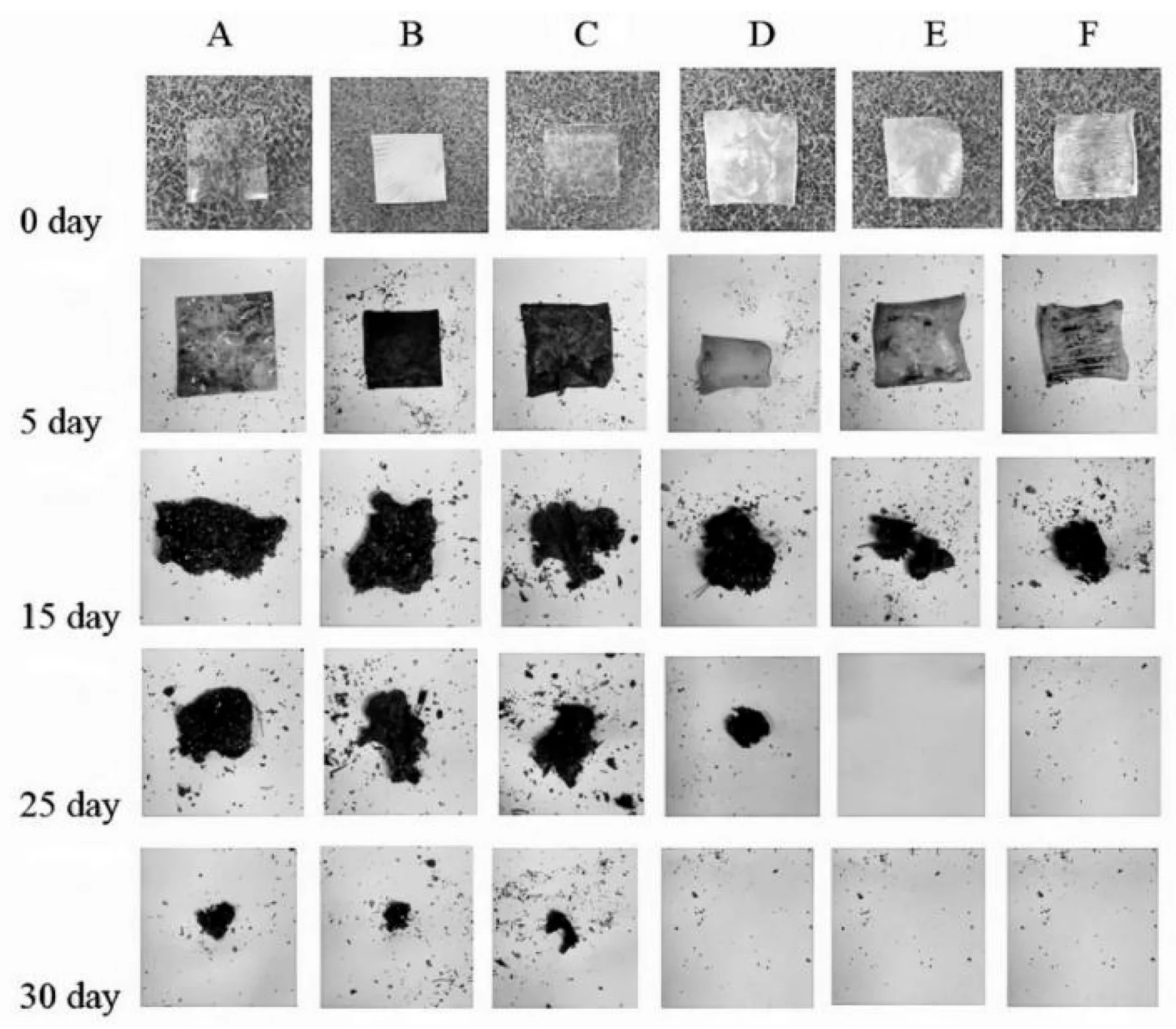
Photographs of composite films after 30 days of soil degradation: (**A**) Neat PVA; (**B**) AX/PVA base film; (**C**) 4% TS/PVA; and (**D**–**F**) 4%, 6%, and 8% TS/AX/PVA films.

**Figure 5:**
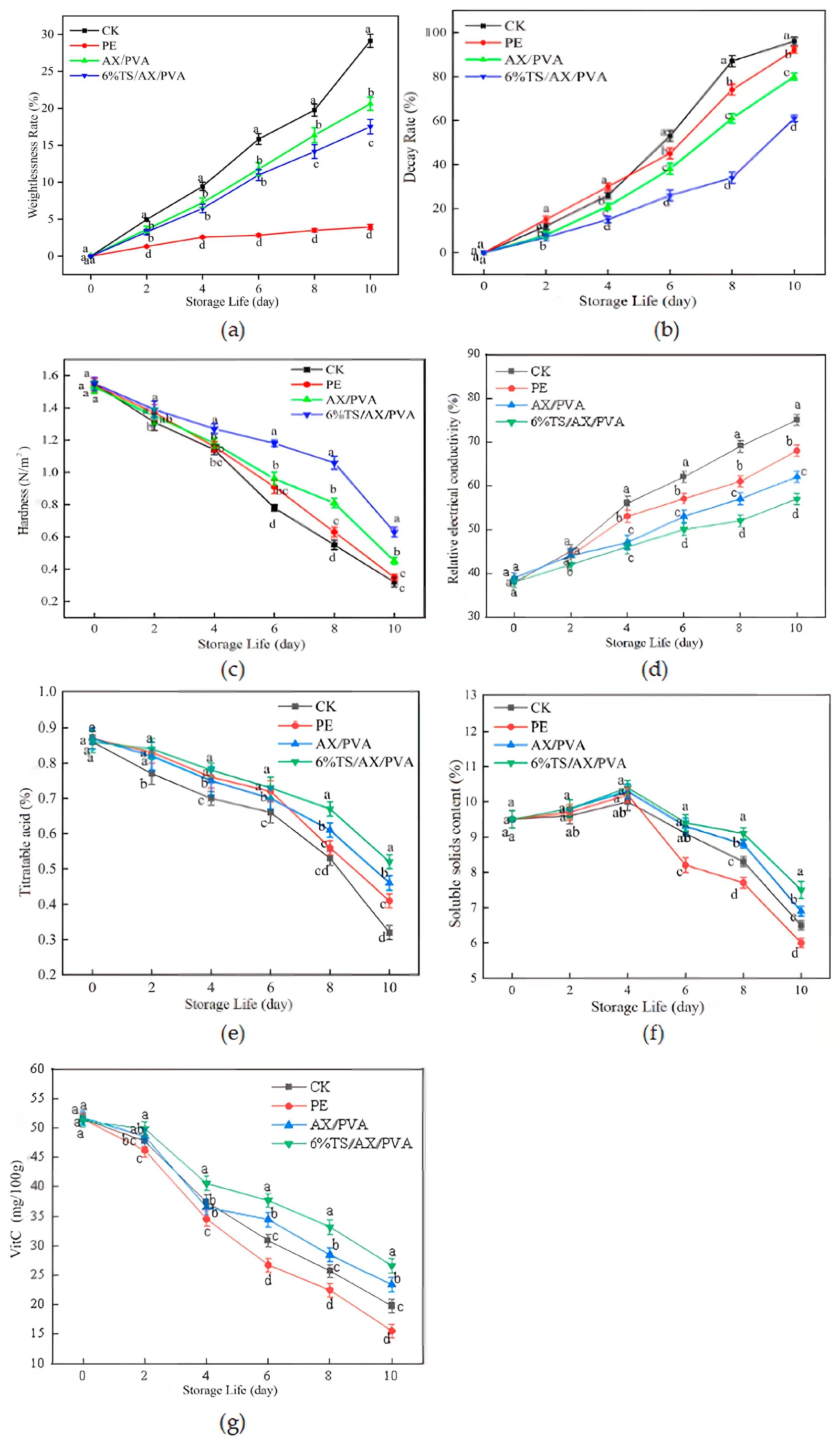
Effects of different composite films on physiological and quality indexes of fresh strawberries: (**a**) Weight loss rate, (**b**) decay rate, (**c**) hardness, (**d**) relative conductivity, (**e**) titratable acidity, (**f**) soluble solids content, and (**g**) VitC content. Values followed by different lowercase letters are significantly different at *p* < 0.05 according to Duncan’s multiple-range test.

**Figure 6:**
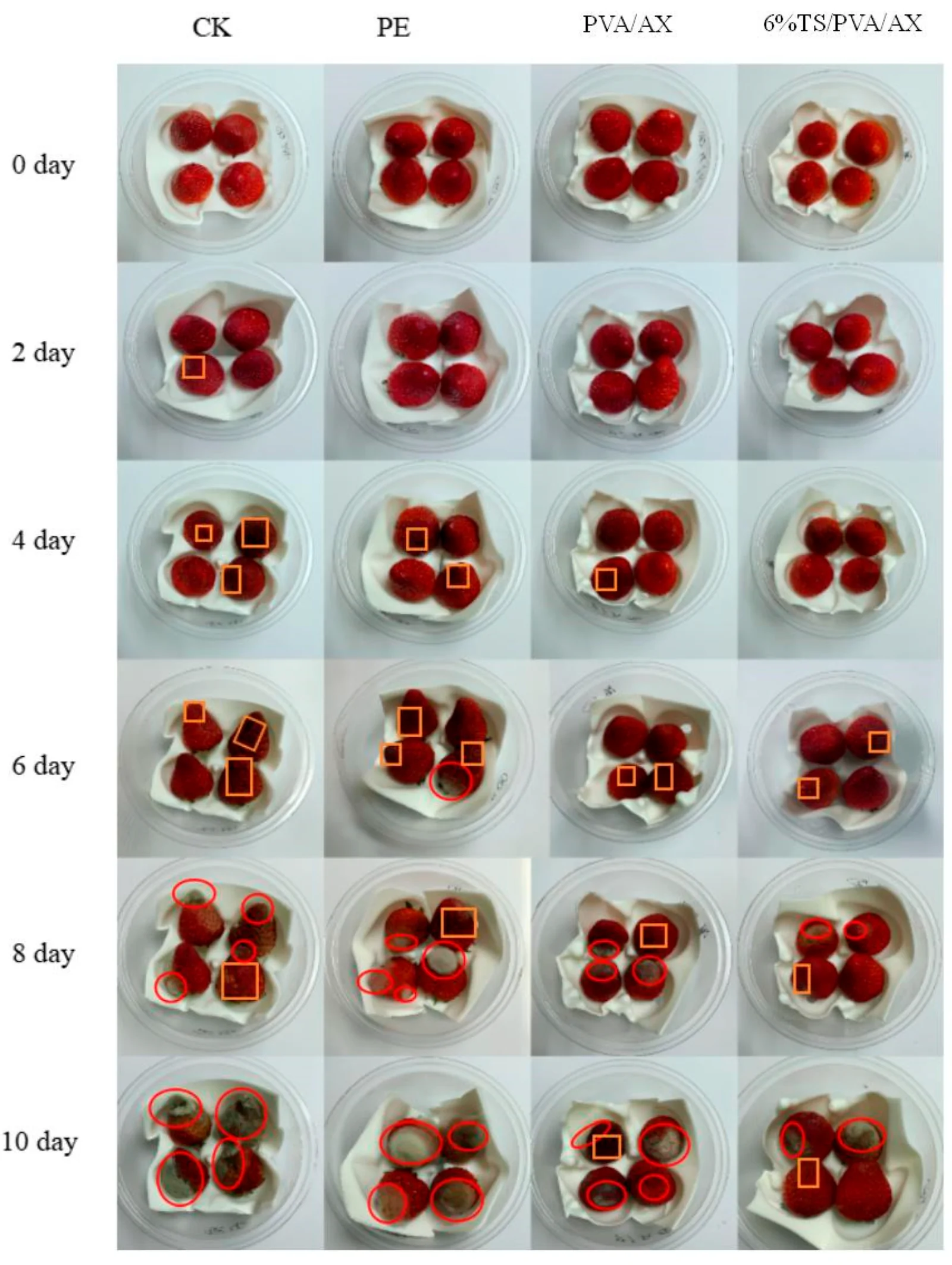
Effects of different composite films on external quality of fresh strawberries (orange square, flesh sagging and pericarp darkening; red circle, surface white plaques and fungal growth).

**Figure 7:**
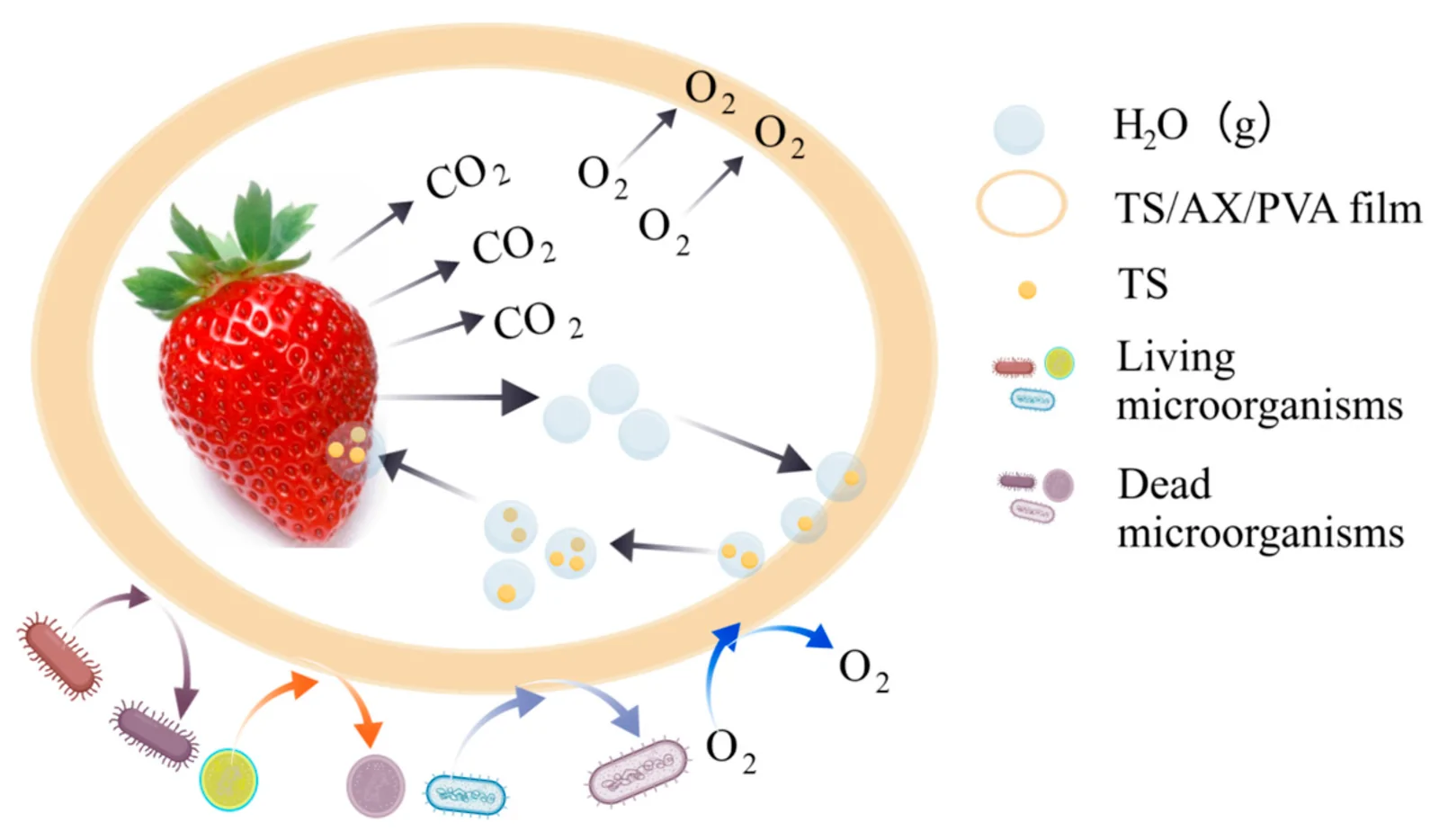
Schematic of the proposed (hypothetical) preservation mechanism of the composite film for strawberries. The depicted tea-saponin release/migration and headspace O_2_/CO_2_ changes were not experimentally measured and remain to be verified.

**Table 1:** Effect of different films on tensile strength and elongation at break.

Film Sample	Tensile Strength (MPa)	Elongation at Break (%)
PVA	29.18 ± 1.26	258.23 ± 4.81
AX/PVA	18.23 ± 2.31	278.41 ± 5.83
4%TS/PVA	14.68 ± 1.67	289.85 ± 8.46
4%TS/AX/PVA	15.54 ± 0.97	314.31 ± 7.22
6%TS/AX/PVA	14.24 ± 2.48	330.81 ± 4.98
8%TS/AX/PVA	10.72 ± 3.29	306.64 ± 6.32

The proportion of AX and PVA is 2 to 8.

**Table 2:** Effect of different films on thickness, moisture content, and water solubility.

Film Sample	Thickness (mm)	Moisture Content (%)	Water Solubility (%)
PVA	0.055 ± 0.007 ^f^	23.95 ± 0.082 ^a^	0.62 ± 0.013 ^a^
AX/PVA	0.061 ± 0.005 ^d^	13.45 ± 0.046 ^d^	0.48 ± 0.008 ^c^
4%TS/PVA	0.064 ± 0.005 ^c^	17.32 ± 0.025 ^c^	0.53 ± 0.011 ^b^
4%TS/AX/PVA	0.069 ± 0.005 ^c^	19.71 ± 0.018 ^b^	0.46 ± 0.036 ^d^
6%TS/AX/PVA	0.077 ± 0.006 ^b^	10.39 ± 0.023 ^e^	0.35 ± 0.039 ^f^
8%TS/AX/PVA	0.085 ± 0.005 ^a^	10.67 ± 0.031 ^e^	0.41 ± 0.032 ^e^

The data are presented as mean ± standard deviation, and different superscript letters indicate significant difference (*p* < 0.05) within the same column. The proportion of AX and PVA is 2 to 8.

## Data Availability

The original contributions presented in this study are included in the article/Supplementary Material. Further inquiries can be directed to the corresponding authors.

## Supplementary Materials

The following supporting information can be downloaded at https://www.mdpi.com/article/10.3390/foods15193421/s1. References [57,58,59,60,61,62,63,64,65,66,67,68,69,70,71,72,73,74] are cited in the Supplementary Materials.
