# Supplementary material for: Biodegradable Tea Saponin/Xylan/PVA Composite Films with Antibacterial and Antioxidant Properties for Strawberry Preservation
Source: Foods. 2026 Sep 24;15(19):3421. doi: 10.3390/foods15193421 (PMC13664123; doi:10.3390/foods15193421)
Supplement: Supplementary file 1 [file foods-15-03421-s001.zip › foods-4521350-supplementary.pdf]

Table of Contents

S1. Ultrasonic-Assisted Aqueous Extraction Process and Property Characterization of TS................2
1.1 Experimental Materials and Apparatus..2
1.1.1 Experimental Materials.. 2
1.1.2 Raw Material Pretreatment...2
1.1.3 Ultrasonic-Assisted Aqueous Extraction of TS and Yield Calculation....................... 2
1.1.4 Single-Factor Experiment for TS Extraction..2
1.1.5 Orthogonal Experiment for TS Extraction.. 3
1.1.6 Decolorization and Purification Process for Crude TS..3
1.1.7 Study on the Properties of Purified TS...4
1.1.8 Data Processing Methods...5
S2. Results and Analysis...5
2.1 Single-Factor Experiment..5
2.2 Optimization of Ultrasonic-Assisted Hot Water Extraction Process for TS.................8
2.3 Decolorization and Purification Process of TS...8
2.4 Characterization of Decolorized TS... 12
S3. Optimization of Xylan Extraction from Camellia oleifera Shell Residues via Acid-Alkali Combination Method and Its Decolorization Process...13
3.1 Experimental Materials and Apparatus..13
3.1.1 Experimental Materials.. 13
3.1.2 Single-Factor Experimental Design for Xylan Extraction from Camellia oleifera Shell Fiber Residues..13
3.1.3 Optimization of Xylan Extraction Process from Camellia oleifera Shell Fiber Residue via Orthogonal Experiment.. 13
3.1.4 Experimental Study on Desalination and Decolorization of Xylan Solution Using Ion Exchange Resin.. 14
3.1.5 Fourier Transform Infrared (FT-IR) Spectroscopic Analysis of Camellia oleifera Shell Xylan Powder... 15
3.1.6 Electron Microscopy Analysis and Particle Size Determination of Camellia oleifera Shell Xylan Powder..15
3.1.7 Nuclear Magnetic Resonance (NMR) Analysis of Camellia oleifera Shell Xylan Powder...15
3.1.8 Data Processing Method.. 16
3.2 Results and Analysis..16
3.2.1 Effect of Different Extraction Conditions on Xylan Yield...16
3.2.2 Optimization of Process Parameters via Orthogonal Experimental Design........... 17
3.2.3 Screening of Different Single Resins for Static Decolorization and Desalination of Xylan Solution.. 18
3.2.4 Screening of Different Resin Combinations for Static Decolorization and Desalination of Xylan Solution..19
3.2.5 Optimization of Decolorization and Purification Process Using D151 + D301 Composite Resin.. 21
3.2.6 Xylan Analysis and Characterization..24

S4. The strawberry sampling scheme for each treatment group..27
S5. Effect of AX/PVA volume ratio on tensile strength & elongation at break..................................28

1. Ultrasonic-Assisted Aqueous Extraction Process and Property Characterization of TS

1.1 Experimental Materials and Apparatus

1.1.1 Experimental Materials

Sodium hydroxide and sulfuric acid were obtained from Beijing Chemical Factory. Vanillin and α-naphthol were supplied by Shanghai Macklin Biochemical Technology Co., Ltd. Aluminum sulfate was purchased from Shanghai Jizhi Biochemical Technology Co., Ltd. Anhydrous ethanol was provided by Modern Orient (Beijing) Technology Development Co., Ltd. A 1 mg/mL aqueous solution of hydrogen peroxide was obtained from Beijing Merida Technology Co., Ltd. DPPH (analytical grade) was sourced from Sigma-Aldrich Chemical Co., USA. LB medium (biological grade) was supplied by Beijing Aobosun Biotechnology Co., Ltd. All chemicals were of analytical grade and used without further purification.

1.1.2 Raw Material Pretreatment

The outer pericarp of the *Camellia oleifera* fruit was thoroughly rinsed with distilled water to remove suspended particulates and dust. Subsequently, the cleaned samples were dried at 60 °C until a constant mass was attained, followed by pulverization. Given that the fruit shell exhibits a more compact structure with high cellulose and lignin contents — where oil is primarily localized in the epidermal layer or intercellular spaces—extraction is inherently more difficult, often resulting in low oil yield [57].

In contrast to the oil-rich *Camellia oleifera* cake meal, the shell itself contains almost no oil, underscoring the need to target other high-value components such as tea saponins.

1.1.3 Ultrasonic-Assisted Aqueous Extraction of TS and Yield Calculation

In this study, ultrasonic technology was combined with distilled water as the solvent to efficiently extract tea saponins from *Camellia oleifera* shells. Accurately weighed 3.00 g samples of shell powder, sieved through a 60-mesh screen, were placed into 50 mL beakers. Ultrasonic extraction was then performed under varying conditions of temperature, solid-liquid ratio, duration, and pH. Following extraction, the tea saponin-containing extract was separated via centrifugation. The preparation of the tea saponin standard solution and the plotting of the standard curve were conducted following the method described by Du [58]. A 0.5 mL aliquot of the supernatant from the diluted extract was taken, and the procedure was identical to that used for the standard curve. The resulting standard curve equation was: $y = 0.6354x + 0.0006$ (where y represents absorbance and x denotes the mass concentration of tea saponin in mg/mL), with a correlation coefficient $R^2 = 0.9982$. The yield of tea saponin was calculated using Equation (S1-1):

$$\text{Extraction Yield of Tea Saponin}(\%) = \frac{nCV}{M} \times 100 \qquad \text{(S1-1)}$$

In Equation (S1-1), n represents the dilution factor; C is the mass concentration of saponin (mg/mL); V is the volume of the sample solution (mL); and M is the mass of *Camellia oleifera* shell (mg).

1.1.4 Single-Factor Experiment for TS Extraction

To systematically evaluate and quantify the effect of individual parameters on extraction efficiency, independent stepwise optimization experiments were conducted for four key factors: extraction temperature, liquid-to-solid ratio, ultrasonic treatment duration, and pH. The factor design levels are presented in Table S1.

Table S1 Single-Factor Experimental Design

Level	Factor			
	**A: Extraction temperature (°C)**	**B: Liquid-to-solid ratio(g/mL)**	**C: PH**	**D: Ultrasonic treatment duration(min)**
1	60	1:05	6	20
2	65	1:10	7	25
3	70	1:15	8	30
4	75	1:20	9	35
5	80	1:25	10	40

1.1.5 Orthogonal Experiment for TS Extraction

Based on the results of the single-factor experiments, an orthogonal experiment was designed to systematically explore and determine the optimal comprehensive conditions for tea saponin extraction. Extraction temperature, liquid-to-solid ratio, ultrasonic treatment duration, and solution pH were identified as the key variables. The parameter ranges for the orthogonal array were defined by referencing the influence patterns of each variable on extraction efficiency, with the level center determined from the optimal condition screening results of the preliminary single-factor experiments. Expansions toward both extremes were then made to construct the corresponding orthogonal experimental design.

1.1.6 Decolorization and Purification Process for Crude TS

To eliminate non-target components (e.g., tannins, proteins, and waxes) from the extraction system, aluminum sulfate was employed as a flocculant for pretreatment of the extract. Following solid-liquid separation via vacuum filtration, the resulting clear filtrate was transferred to a constant-temperature magnetic stirrer and concentrated under reduced pressure at 70–80 °C to one-fifth of its original volume. This pre-concentrated product was then transferred to a separatory funnel, and anhydrous ethanol was added at a volume ratio of 1:3 for three consecutive extractions.

The upper organic phases were collected and combined. Ethanol was evaporated at 50 °C, and the residue was frozen. After vacuum freeze-drying, crude tea saponin samples were obtained. A precise 10.00 mg aliquot of the sample was weighed, dissolved in anhydrous ethanol, and diluted to a final volume of 10.0 mL. A standard curve was constructed using the vanillin-concentrated sulfuric acid colorimetric method, and the exact mass concentration of tea saponin in the test solution was calculated via the regression equation. The purity of the crude tea saponin was determined using Equation (S1-2):

$$\text{Purity of Crude Tea Saponin}(\%) = \frac{c_1}{c_2} \times 100 \quad \text{(S1-2)}$$

In Equation (S1-2), C_1 represents the mass concentration of crude Camellia saponin (mg/mL), and C_2 represents the mass concentration of Camellia saponin in the initial shell extract (mg/mL).

The crude extract obtained via preliminary separation not only contains co-dissolved impurities such as proteins, polysaccharides, and polyphenolic compounds, but also exhibits coloration from endogenous pigments. This issue not only degrades the product's appearance but also induces negative sensory evaluation outcomes, severely limiting its application potential in refined fields such as high-end cosmetics and food-grade surfactants. To address this, a 2.00 wt% aqueous solution of tea saponin was prepared to investigate the effects of

varying H_2O_2 dosages (15, 20, 25, 30%), decolorization temperatures (55, 60, 65, 70 °C), pH values (7, 8, 9, 10), and treatment durations (30, 60, 90, 120 min) on decolorization performance. Absorbance values before and after decolorization were measured at the maximum absorption wavelength determined using an ultraviolet-visible (UV-Vis) spectrophotometer. The decolorization rate and tea saponin loss rate were calculated using Equations (S1-3) and (S1-4), respectively.

$$\text{Decolorization rate}(\%) = \frac{x_1 - x_2}{x_1} \times 100 \qquad \text{(S1-3)}$$

In Equation (S1-3), X_1 represents the absorbance of the solution before decolorization and X_2 represents the absorbance after decolorization. The yield of tea saponin before and after decolorization was measured by the method described in Section 1.1.3, and the loss rate was calculated.

$$\text{Tea saponin loss rate}(\%) = \frac{T_1 - T_2}{T_1} \times 100 \qquad \text{(S1-4)}$$

In Equation (S1-4), T_1 represents the yield of tea saponin in the solution before decolorization (%), and T_2 represents the yield after decolorization (%).

The solution decolorized and purified with $H_2O_2$was concentrated and then subjected to vacuum freeze-drying to yield a high-purity tea saponin sample. A precisely weighed 10.00 mg portion of the sample was dissolved in anhydrous ethanol and quantitatively transferred to a 10.0 mL volumetric flask.

A standard curve was constructed using the vanillin-concentrated sulfuric acid colorimetric method, and the exact mass concentration of tea saponin in the test solution was calculated via the regression equation. The purity of the final tea saponin sample was determined using Equation (S1-5):

$$\text{Purity of the tea saponin sample}(\%) = \frac{C_3}{C_1} \times 100 \qquad \text{(S1-5)}$$

In Equation (S1-5), C_1represents the mass concentration of crude tea saponin (mg/mL), and C_3 represents the mass concentration of decolorized tea saponin (mg/mL).

1.1.7 Study on the Properties of Purified TS

(1) Fourier Transform Infrared (FTIR) Spectroscopy Analysis

The purified sample was subjected to Fourier Transform Infrared (FTIR) spectroscopy scanning to identify its characteristic functional groups.

(2) Color Reaction

The characteristic color reaction between tea saponin and naphthol was verified, with experimental procedures performed in accordance with Reference [59].

(3) Determination of Antioxidant Properties

The DPPH radical scavenging capacity of tea saponin at varying mass concentrations (0.25–4 mg/mL) was measured following the method established by Feng Yanru [60].

(4) Antibacterial Assay

The Oxford cup method was employed to evaluate the antibacterial activity of the test samples. A series of tea saponin solutions with gradient mass concentrations (mg/mL) were prepared according to the experimental design. The procedures for activating *Escherichia coli* and *Staphylococcus aureus* were conducted[61]. A 100 μL aliquot of the diluted bacterial suspension was accurately pipetted and evenly spread onto the culture medium using a sterile spreader to ensure uniform microbial distribution. Subsequently, three sets of sterilized Oxford cups were precisely placed in the Petri dish with sterile forceps, maintaining equidistant spacing. Each Oxford cup was then filled with 20 μL of the tea saponin solution, and the Petri

dishes were incubated at 4 °C for 3–4 h to facilitate solution diffusion. The plates were then incubated upright at 37 °C for 24 h, after which the diameters of the inhibition zones were measured and recorded.

1.1.8 Data Processing Methods

Statistical analysis of the data was performed using Origin 2018 and SPSS 26.0 software. Results with $p < 0.05$ were considered statistically significant. Different lowercase letters (a, b, c, d) denote significant differences between corresponding groups, where the order of the letters reflects the ranking of the mean values. No statistically significant differences were observed between groups labeled with the same letter.

2. Results and Analysis

2.1 Single-Factor Experiment

(1) Effect of Extraction Temperature on the Extraction Yield of TS

Under fixed conditions of a liquid-to-solid ratio of 1:11 (mL:g), pH 8.0, and an ultrasonic extraction duration of 30 min, the effect of extraction temperature (60, 65, 70, 75, and 80 °C) on the yield of tea saponin was systematically investigated. The experimental results revealed that the extraction yield exhibited a trend of first increasing and then decreasing with rising temperature. As shown in Figure S1, the solubility of tea saponin in hot water increased significantly when the temperature was elevated, indicating its favorable temperature-dependent solubility.

The maximum extraction yield (12.71%) was achieved at 70 °C. However, when the temperature exceeded 70 °C, two main effects were observed: an accelerated leaching of polysaccharide components from *Camellia oleifera* shells, which may form complexes with partial tea saponin and induce precipitation; and a marked increase in the gelatinization rate of water-soluble starch, leading to elevated solution viscosity. This not only increased the difficulty of tea saponin separation but also posed challenges to subsequent processing, ultimately resulting in a decrease in tea saponin concentration in the extract [62]. Therefore, the initial extraction temperature was tentatively set at 70 °C.

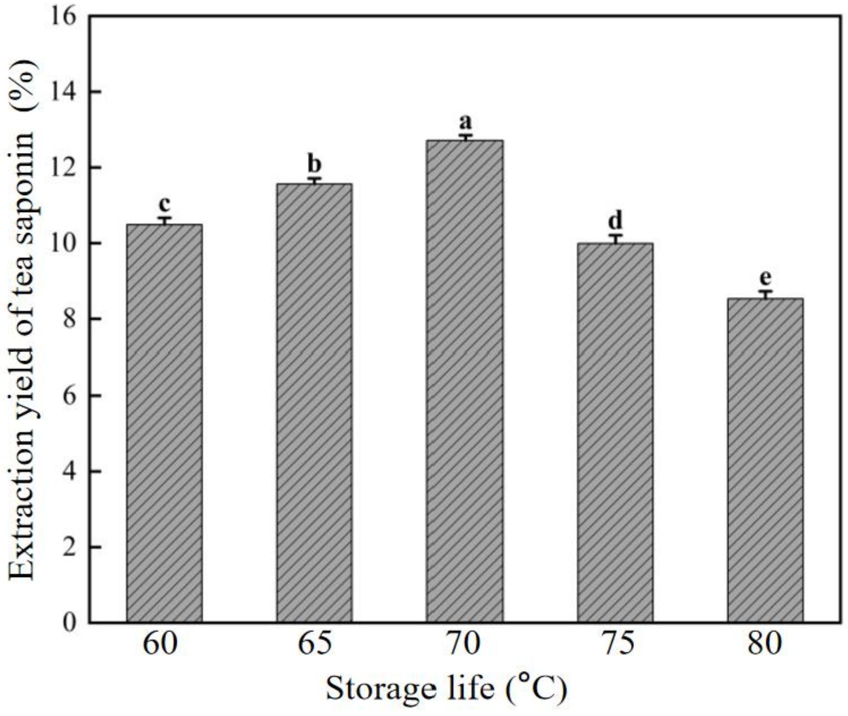

Fig. S1. Effect of extraction temperature on the extraction rate of tea saponin

(2) Effect of Liquid-to-Solid Ratio on the Extraction Yield of TS

Under conditions of pH 8.0, an ultrasonic extraction duration of 30 min, and an extraction temperature of 70 °C, the effect of different liquid-to-solid ratios on the extraction yield of tea saponin was investigated. The results are presented in Figure S2. Within the tested range of 1:5 to 1:17 (m/v), the extraction yield first increased and then gradually decreased. When the liquid-to-solid ratio reached 1:14, the extraction yield peaked at 12.66%. A low extraction yield at low liquid-to-solid ratios may be attributed to insufficient extraction solvent, leading to slow molecular diffusion. Conversely, at relatively high liquid-to-solid ratios, the solute concentration decreases, which expands the contact area between the solute and the material, thereby enhancing the driving force for mass transfer and correspondingly increasing the extraction rate [63]. When the liquid-to-solid ratio exceeded 1:11, the change in extraction yield became negligible. An increase in solvent dosage dilutes the solute concentration in the extract. While this may reduce energy consumption during the subsequent concentration step, it also promotes the dissolution of more non-target impurities, thereby raising overall process costs [64]. Considering these factors, the optimal liquid-to-solid ratio was determined to be 1:11.

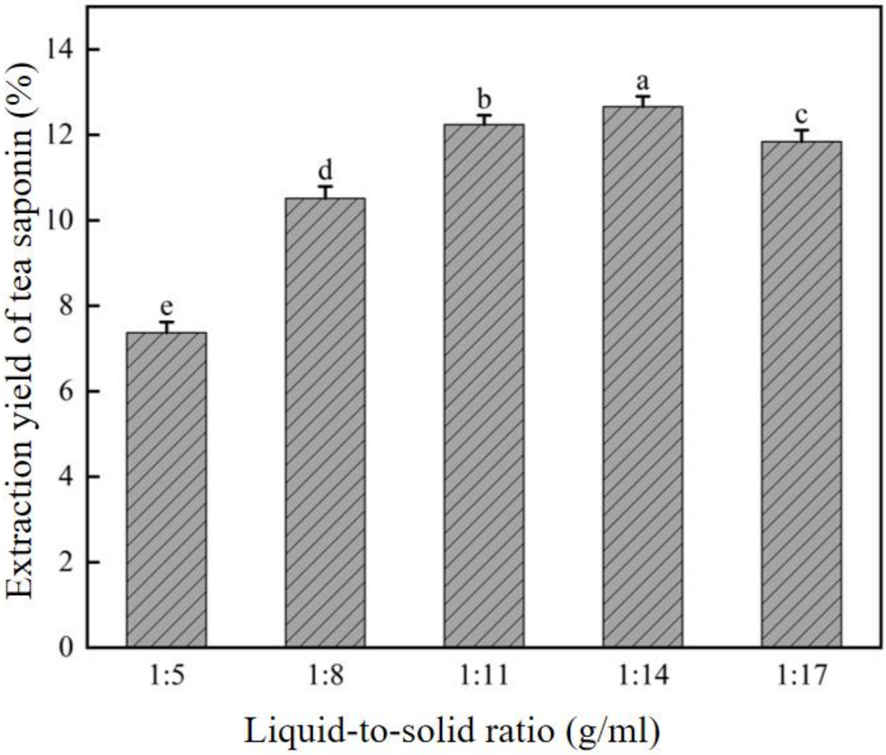

Fig. S2. Effect of liquid-to-solid ratio on the extraction yield of tea saponin

(3) Effect of Extraction Time on TS Extraction Yield

Under fixed conditions of a liquid-to-solid ratio of 1:11 (mL:g), pH 8.0, and an extraction temperature of 70 °C, the influence of extraction time on tea saponin extraction efficiency was systematically investigated. The detailed extraction yield data are presented in Figure S3.

With prolonged ultrasonic extraction, the dissolution rate of tea saponins initially increased. However, once the concentration of tea saponins in the extract reached saturation, further extension of ultrasonic time failed to enhance the extraction yield. Theoretically, an optimal ultrasonic duration exists to balance extraction efficiency and economic feasibility [65]. In this study, the extraction efficiency of tea saponins peaked at an ultrasonic duration of 30 min, indicating that most target components had been effectively extracted by this time point. No significant increase in yield was observed with longer extraction times. After comprehensive consideration of the extraction cycle and energy consumption, the optimal ultrasonic duration was determined to be 30 min.

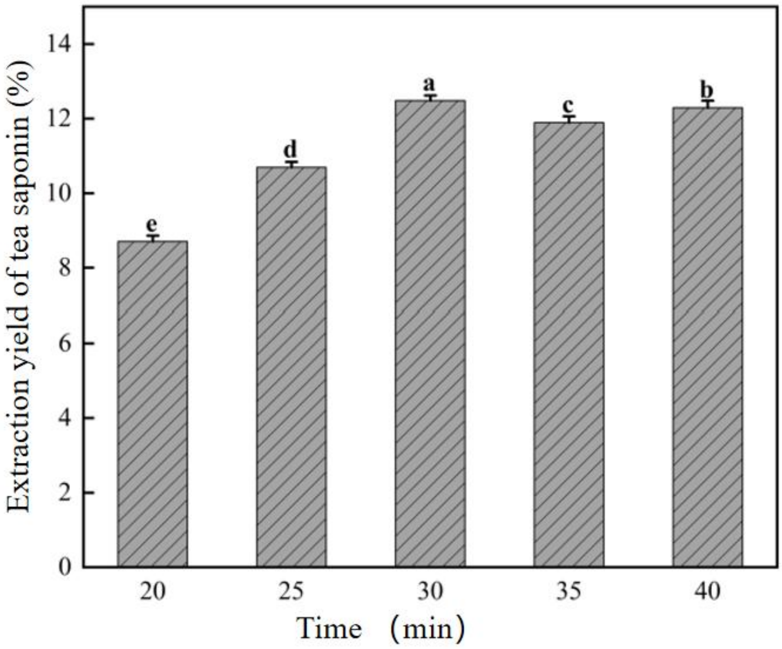

Fig. S3. Effect of extraction time on the extraction yield of tea saponin

(4) Effect of pH on TS Extraction Yield

Under fixed conditions of a liquid-to-solid ratio of 1:11 (mL :g), an extraction temperature of 70 °C, and an ultrasonic duration of 30 min, the effect of pH on tea saponin extraction efficiency was systematically evaluated. Detailed extraction yield data are provided in Figure S4.

The extraction efficiency of tea saponins exhibited a trend of first increasing and then decreasing with rising pH, peaking at 12.35% when the pH was adjusted to 9.0. Given the weakly acidic nature of tea saponins, a moderately alkaline environment is critical for facilitating their dissolution [58]. However, in strongly alkaline conditions (pH > 9.0), alkali-soluble impurities were solubilized and potentially formed complexes with tea saponins, leading to a significant decline in extraction efficiency. Consistent with previous reports, tea saponins show reduced stability in strongly alkaline environments, which may result in a marked decrease in their concentration in the extract.

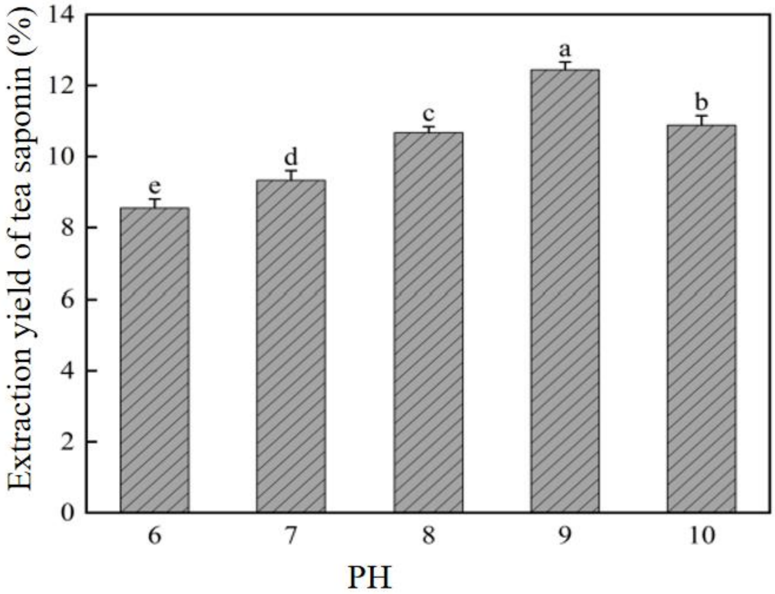

Fig. S4. Effect of pH on the extraction yield of tea saponin

2.2 Optimization of Ultrasonic-Assisted Hot Water Extraction Process for TS

The factors and levels for the orthogonal experiment designed to optimize the ultrasonic-assisted hot water extraction of tea saponin are presented in Table S2.

Table S2 Factors and levels for the orthogonal experiment of ultrasound-assisted aqueous extraction of tea saponin

Level	Factor			
	**A: Extraction temperature (°C)**	**B: Liquid-to-solid ratio(g/mL)**	**C: PH**	**D: Ultrasonic treatment duration(min)**
1	65	1:08	8	25
2	70	1:11	9	30
3	75	1:14	10	35

This study systematically investigated the regulatory effects of different factors on tea saponin extraction performance. By integrating orthogonal experimental design with range analysis, the contribution of each key parameter to extraction efficiency was quantified, and the optimal combination of process parameters was thereby determined. Relevant experimental data and analysis results are detailed in Table S3.

Table S3 Orthogonal test results for ultrasound-assisted aqueous extraction of tea saponin

Run Number	Factor				TS Extraction Yield (%)
	**A**	**B**	**C**	**D**	
1	1	1	1	1	11.19
2	1	2	2	2	10.88
3	1	3	3	3	11.08
4	2	1	2	3	11.53
5	2	2	3	1	12.11
6	2	3	1	2	12.78
7	3	1	3	2	11.04
8	3	2	1	3	10.67
9	3	3	2	1	10.34
K1	33.15	33.76	34.64	33.64	
K2	36.42	33.66	32.75	34.7	
K3	32.05	34.2	34.23	33.28	
k1	11.05	11.25	11.55	11.21	
k2	12.14	11.22	10.92	11.57	
k3	10.68	11.40	11.41	11.09	
R	1.46	0.18	0.63	0.47	
Factor Priority Ranking	A>C>D>B				
Optimal Condition Combination	A2	B3	C1	D2	

According to the range analysis data presented in Table S3, the key factors influencing tea saponin extraction efficiency, ranked by their significance, are as follows: extraction temperature (A) > extraction pH (C) > extraction time (D) > liquid-to-solid ratio (B). This finding indicates that extraction temperature is the most critical parameter to control, followed by pH and time, whereas the liquid-to-solid ratio has a relatively minor impact on the extraction yield. Based on the experimental results, the optimal combination of process parameters was identified as $A_2B_3C_1D_2$, corresponding to an extraction temperature of 70 °C, a liquid-to-solid ratio of 1:14, an extraction pH of 8.0, and an ultrasonic duration of 30 min. Under these optimized conditions, the tea saponin yield reached 12.78%, validating the effectiveness of this parameter combination.

2.3 Decolorization and Purification Process of TS

(1) Effect of H_2O_2Dosage on TS Loss Rate and Decolorization Efficiency

Owing to its strong oxidizing capacity and high thermal stability, hydrogen peroxide (H_2O_2) is widely utilized in the food and beverage industries. Decolorization of tea saponin occurs when free radicals generated from H_2O_2 react chemically with pigments in the aqueous solution [66]. Under fixed conditions of 65 °C, a decolorization pH of 9.0, and a decolorization time of 60 min, the effects of varying H_2O_2 dosages on tea saponin loss rate and decolorization efficiency were investigated. The results are presented in Figure S5.

Both the tea saponin loss rate and decolorization efficiency increased with increasing H_2O_2 dosage. This is because pigments typically contain conjugated double bonds. As an oxidative decolorizing agent, H_2O_2 disrupts the chromophoric groups of pigment molecules by inducing cleavage or isomerization of these conjugated double bonds, thereby achieving decolorization [67]. It was observed that when the H_2O_2 dosage exceeded 25%, the decolorization efficiency gradually plateaued, whereas the tea saponin loss rate continued to rise. This phenomenon is primarily attributed to the presence of reactive groups (e.g., hydroxyl groups) in tea saponin; an excessive H_2O_2 dosage may lead to increased degradation of the tea saponin itself. Thus, at an H_2O_2 dosage of 25%, the decolorization efficiency reached 81.59%, while the tea saponin loss rate was 19.23%.

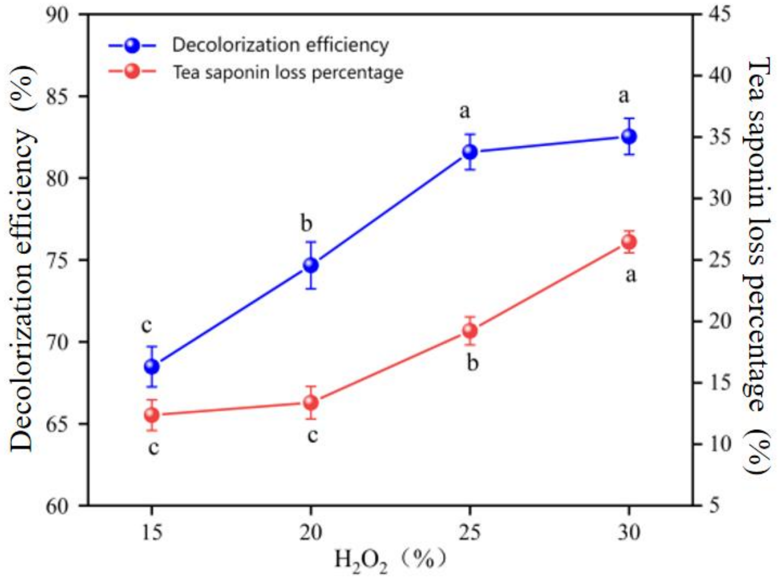

Fig. S5. Effect of H_2O_2 dosage on tea saponin loss rate and decolorization rate

(2) Effect of Temperature on TS Loss Rate and Decolorization Efficiency

Under the conditions of 25% H_2O_2 addition, a decolorization pH of 9.0, and a decolorization duration of 60 min, the effects of varying decolorization temperatures on tea saponin loss rate and decolorization efficiency were investigated. The results are presented in Figure S6.

As the decolorization temperature increased from 55 °C to 65 °C, decolorization efficiency increased significantly, reaching 75.14% at 65 °C. Beyond 65 °C, however, decolorization efficiency gradually declined. This phenomenon can be attributed to the decomposition rate of H_2O_2 exceeding the oxidation rate of pigments, leading to a sharp decrease in free radical concentration and thus reduced decolorization performance [68]. Concurrently, within the temperature range of 55–70 °C, the tea saponin loss rate exhibited a continuous upward trend, peaking at 32.18% at 70 °C. Two underlying mechanisms explain this observation. First, tea saponin is inherently thermosensitive, and elevated temperatures may induce cleavage of its glycosidic bonds. Second, high temperatures accelerate H_2O_2 decomposition, generating excessive highly reactive hydroxyl radicals. While these radicals effectively oxidize pigments,

surplus radicals non-selectively attack reactive groups in tea saponin molecules, causing structural disruption and ultimately increasing the loss rate. Comprehensively balancing tea saponin retention, decolorization efficiency, and energy consumption, the optimal decolorization temperature was determined to be 65 °C.

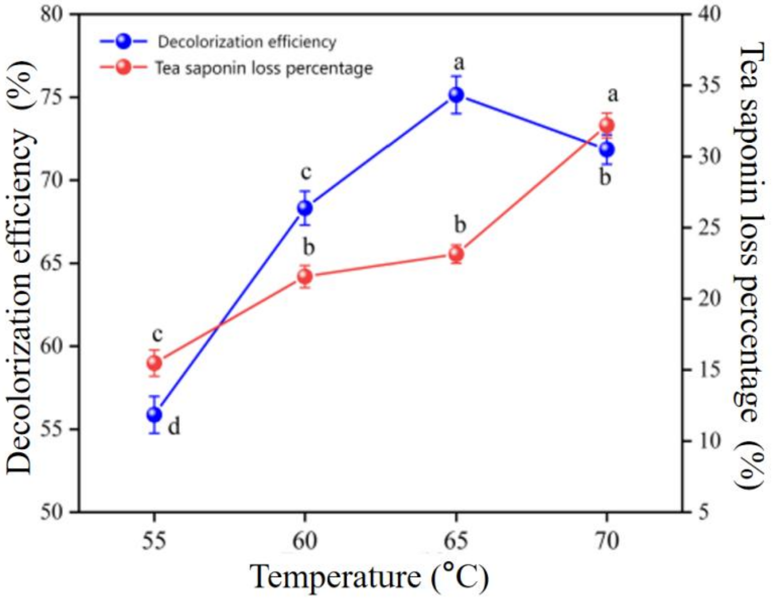

Fig. S6. Effect of temperature on tea saponin loss rate and decolorization rate

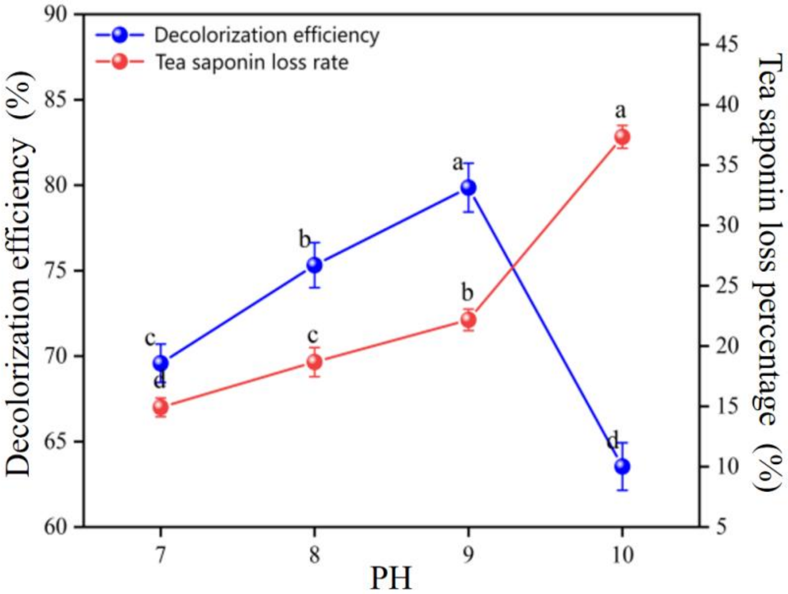

Fig. S7. Effect of pH on tea saponin loss rate and decolorization rate

(3) Effect of pH on TS Loss Rate and Decolorization Efficiency

Under the conditions of 25% H_2O_2 addition, a decolorization temperature of 65 °C, and a decolorization duration of 60 min, the effects of varying decolorization pH values on tea saponin loss rate and decolorization efficiency were investigated. The results are presented in Figure S7.

The decolorizing activity of H_2O_2 in aqueous solutions is primarily mediated by the hydroperoxyl anion (HO_2^-). In both acidic and alkaline environments, H_2O_2 undergoes activation to facilitate HO_2^- generation; thus, solution pH directly modulates decolorization performance, with alkaline conditions being more favorable for HO_2^- formation. As depicted in the figure, within the alkaline pH range of 7.0–10.0, decolorization efficiency first increased

and then decreased, peaking at 79.86% at pH 9.0. When the pH exceeded 9.0, decolorization efficiency declined significantly. This is attributed to the accelerated decomposition of H_2O_2 into water and oxygen under excessively alkaline conditions, which increases the proportion of ineffective decomposition, reduces free radical production, and consequently diminishes oxidative capacity [69]. The tea saponin loss rate increased gradually within the pH range of 7.0–9.0, reaching 22.17% at pH 9.0. A sharp rise in loss rate was observed at pH 10.0, indicating that the saponin structure remains relatively stable under neutral to weakly alkaline conditions, whereas a strongly alkaline environment may induce cleavage of chemical bonds in tea saponin molecules, leading to irreversible degradation. Comprehensively balancing tea saponin retention and decolorization efficiency, the optimal decolorization pH was determined to be 9.0.

(4) Effect of Decolorization Time on TS Loss Rate and Decolorization Efficiency

Under the conditions of 25% H_2O_2 addition, a decolorization temperature of 65 °C, and a decolorization pH of 9.0, the effects of varying decolorization durations on tea saponin loss rate and decolorization efficiency were investigated. The results are presented in Figure S8.

As decolorization time extended, both tea saponin loss rate and decolorization efficiency exhibited an increasing trend; however, this dynamic plateaued at 60 min. This stabilization is attributed to the substantial reduction in pigment concentration within the system, which diminished the collision frequency between free radicals and pigment molecules. Within the 30–60 min decolorization window, the tea saponin loss rate increased significantly. A plausible mechanism is that as pigment substrates were depleted, free radicals shifted their target to tea saponin molecules—specifically, oxidizing and degrading active groups (e.g., hydroxyl groups, double bonds) within the saponin. When decolorization time exceeded 60 min, the loss rate rose sharply. This may result from either excess H_2O_2 or the prolonged reaction time promoting continuous free radical accumulation, which induces irreversible damage such as molecular chain cleavage and saponin hydrolysis. Furthermore, extending the decolorization duration leads to elevated energy consumption and operational costs. Comprehensively balancing tea saponin retention, decolorization efficiency, and economic feasibility, the optimal decolorization time was determined to be 60 min.

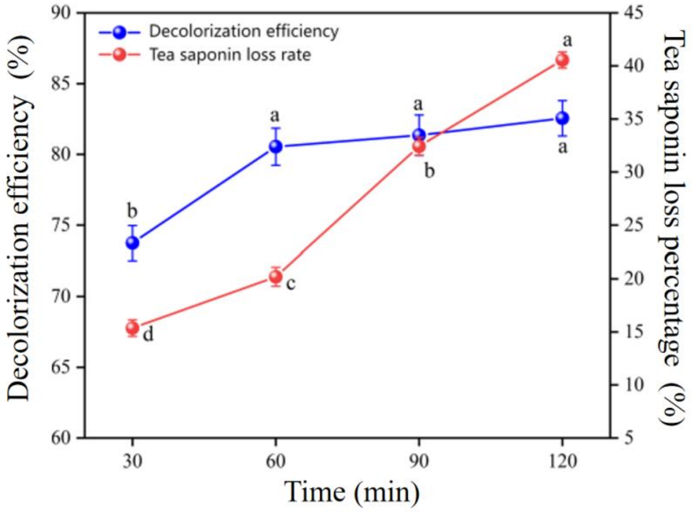

Fig. S8. Effect of decolorization time on tea saponin loss rate and decolorization rate

Based on the experimental results and mechanistic analysis, the optimal decolorization conditions for tea saponin were determined as follows: 25% (v/v) H_2O_2 relative to the volume of the tea saponin aqueous solution, pH adjusted to 9.0, and incubation in a 65 °C water bath for 60 min. Under these optimized conditions, the tea saponin retention rate reached 83.75%, with a decolorization efficiency of 85.46%. The H_2O_2-decolorized and purified tea saponin

solution was subsequently processed through concentration via rotary evaporation, liquid-liquid extraction with 95% ethanol, dialysis, and final vacuum freeze-drying. Following this purification train, the purity of the tea saponin extract was increased from 46.65% to 71.28%.

2.4 Characterization of Decolorized TS

(1) Fourier Transform Infrared Spectroscopic Analysis

The functional groups of the tea saponin aglycone were characterized by FT-IR spectroscopy, as shown in Figure S9. The broad peak at 3425 cm^{-1} corresponds to the characteristic absorption of hydroxyl (O–H) groups. The absorption bands at 2935, 1452, and 1339 cm^{-1} are attributed to the stretching and bending vibrations of methyl and methylene groups. The peak at 1719 cm^{-1} arises from the stretching vibration of aldehyde (C=O) groups, while the signal at 1613 cm^{-1} is assigned to the stretching vibration of C=C bonds. The absorption at 1051 cm^{-1} results from the bending vibration of C–O–H. These characteristic peaks are consistent with those reported for tea saponin extracted via ultrasonic alcohol extraction and ultrasonic water bath extraction in previous studies [70], and they closely match the key spectral features of a tea saponin standard. These data confirm that the extracted substance is indeed tea saponin and demonstrate that the decolorization process did not alter its molecular structure.

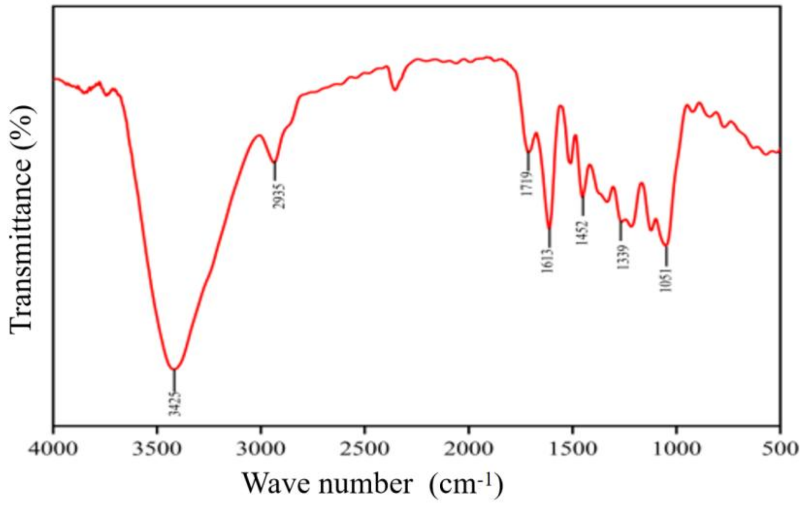

Fig. S9. FT-IR spectrum of tea saponin

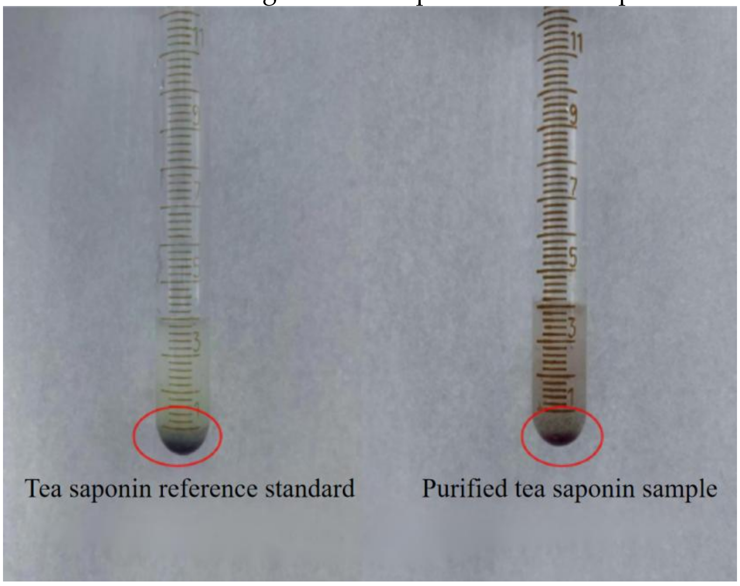

Fig. S10. Molish reaction of tea saponin

(2) Colorimetric Reaction

The characteristic color reaction of tea saponin is shown in Figure S10. A positive Molish reaction was observed, indicated by the formation of a purple ring at the interface between the test solution and the acid layer. This result aligns with the expected outcome for saponins and provides confirmatory evidence that the isolated and identified compound is tea saponin.

3. Optimization of Xylan Extraction from *Camellia oleifera* Shell Residues via Acid-Alkali Combination Method and Its Decolorization Process

3.1 Experimental Materials and Apparatus

3.1.1 Experimental Materials

Hydrochloric acid (HCl), sulfuric acid (H_2SO_4), and sodium hydroxide (NaOH) were purchased from Beijing Chemical Factory. Macroporous adsorption resin D301, 001×7 cation exchange resin, and weakly acidic macroporous resin D151 were obtained from Shanghai Yuanye Biotechnology Co., Ltd. The 717 strong-base type I anion exchange resin was supplied by Shanghai Aladdin Biochemical Technology Co., Ltd. Anhydrous ethanol was procured from Modern Orient (Beijing) Technology Development Co., Ltd. All chemicals were of analytical grade and used without further purification.

3.1.2 Single-Factor Experimental Design for Xylan Extraction from *Camellia oleifera* Shell Fiber Residues

Dried *Camellia oleifera* shell fiber residues (post-tea saponin extraction) were powdered and subjected to mild acid pretreatment with 0.5% (v/v) sulfuric acid at 60 °C for 1 h. This acidic treatment serves dual functions: (1) disrupting the compact lignocellulosic matrix to form a loose, porous structure, and (2) promoting xylan solubilization via proton-catalyzed cleavage of glycosidic linkages in hemicellulose [71].

Using the pretreated residue powder as feedstock, a single-factor experimental design was implemented to evaluate the effects of process parameters on xylan extraction efficiency. The independent variables were: sodium hydroxide (NaOH) concentration (4%, 6%, 8%, 10%, 12% w/v); reaction temperature (70, 75, 80, 85, 90 °C); solid-liquid ratio (1:8, 1:10, 1:12, 1:14, 1:16 g/mL); and reaction duration (1, 2, 3, 4 h). Each parameter was varied individually while others were held constant to elucidate its dose-dependent effect on extraction efficiency.

Post-reaction, the mixture was vacuum-filtered and the residue was washed with deionized water; the combined filtrate and washings were collected. The pH of the aqueous phase was adjusted to 5.5–6.0 using 1 M HCl or NaOH, followed by a 30-min equilibration period. The solution was then centrifuged at 5000 rpm to remove insoluble solids (predominantly cellulose and residual lignin), yielding a xylan-enriched supernatant. Crude xylan was recovered via ethanol precipitation: three volumes of ice-cold anhydrous ethanol were added to the supernatant, and the mixture was incubated at 4 °C for 12 h (or until flocculation ceased). The resulting precipitate was collected by centrifugation (5000 rpm), freeze-dried, and ground to a fine powder. The xylan yield was calculated using Equation (S2-1):

$$\text{Xylan yield}(\%) = \frac{m_1}{m_0} \times 100 \qquad \text{(S2-1)}$$

In Equation (S2-1), m_1 represents the mass (mg) of the dried xylan sample, and m_0 represents the mass (mg) of the *Camellia oleifera* shell fiber residue.

3.1.3 Optimization of Xylan Extraction Process from *Camellia oleifera* Shell Fiber Residue via Orthogonal Experiment

Based on the single-factor experiments, an orthogonal experimental design was adopted to optimize the process parameters for xylan extraction. Sodium hydroxide concentration (8%, 10%, 12%), reaction temperature (75, 80, 85 °C), solid-liquid ratio (1:12, 1:14, 1:16 g/mL), and reaction time (2, 3, 4 h) were selected as the key influencing factors. A four-factor, three-level orthogonal experimental array $L_9(3^4)$ was constructed. The specific experimental scheme is

detailed in Table S4. Through range analysis to determine the significant influence of each factor, the optimal combination of process parameters was identified.

Table S4 Orthogonal Test Factors and Levels

Factor Levels	A : NaOH Solution Concentration(%)	B:Temperature(℃)	C：Solid-Liquid Ratio(g·mL-1)	D:Reaction Time (h)
1	8	75	1: 12	2
2	10	80	1: 14	3
3	12	85	1: 16	4

3.1.4 Experimental Study on Desalination and Decolorization of Xylan Solution Using Ion Exchange Resin

(1) Conventional Acid Precipitation Treatment

The separated filtrate was adjusted to pH 5.5–6.0 using sulfuric acid solution. After a 30-min holding period, the mixture was centrifuged at 5000 rpm for 10 min. An aliquot of the resulting supernatant was subjected to microfiltration through a 0.45 μm membrane. The absorbance of the microfiltered solution was measured at a wavelength of 420 nm, and the decolorization efficiency was calculated in accordance with Equation (S2-2).

$$\text{Decolorization Efficiency}(\%) = \frac{x_3 - x_4}{x_3} \times 100 \qquad \text{(S2-2)}$$

In Equation (S2-2), X_3 represents the absorbance of the test solution before acid precipitation, and X_4 represents the absorbance after acid precipitation.

(2) Decolorization and Desalination of Crude Xylan Extract via Sequential Cation-Anion Exchange Resin Process

Based on a systematic analysis of the decolorization and desalination performance of different ion exchange resins for the crude xylan extract under static adsorption conditions, this study aimed to identify and validate the most effective resin combination. By investigating the synergistic effects between cation and anion exchange resins, the decolorization and desalination process was optimized to determine the optimal purification parameters. Four commonly used ion exchange resins — cation exchange resins (001 × 7, D151) and anion exchange resins (D301, 717)—were selected to compare their decolorization and desalination efficacy for the crude xylan solution.

(3) Static Decolorization Screening of Single Resin Adsorbents

For each pretreated resin, 20 g was weighed and mixed with 60 mL of crude xylan extract for static adsorption at 40 °C for 60 min. The mixture was shaken intermittently every 20 min to facilitate mass transfer. Upon completion of decolorization, solid-liquid separation was performed, and the supernatant was collected to determine three evaluation metrics: decolorization efficiency, desalination rate, and xylan retention rate. These metrics were employed to screen optimal resin adsorbents. The decolorization efficiency, desalination rate, and xylan retention rate were calculated using the following formulas:

$$\text{Decolorization Efficiency}(\%) = \frac{s_1 - s_2}{s_1} \times 100 \qquad \text{(S2-3)}$$

In Equation (S2-3), S_1 represents the absorbance of the sample solution before resin adsorption, and S_2 represents the absorbance after resin adsorption.

$$\text{Desalination Rate}(\%) = \frac{Y_1 - Y_2}{Y_1} \times 100 \qquad \text{(S2-4)}$$

In Equation (S2-4), Y_1 represents the conductivity (μS/cm) of the sample solution before resin adsorption, and Y_2 represents the conductivity after resin adsorption.

$$\text{Cellulose Retention Rate}(\%) = \frac{M_2}{M_1} \times 100 \qquad \text{(S2-5)}$$

In Equation (S2-5), M_1 represents the mass (mg) of the xylan sample before resin adsorption, and M_2 represents the mass after resin adsorption.

(4) Static Decolorization Screening of Different Composite Resins

Four types of anion and cation exchange resins were individually combined at a 1:1 ratio. A 60 mL aliquot of crude xylan extract was added, followed by static adsorption at 40 °C for 60 min with shaking at 20-min intervals. Upon completion of decolorization, solid-liquid separation was performed, and the supernatant was collected to determine and compare the decolorization efficiency, desalination efficiency, and xylan retention rate of xylan solutions treated with different composite resins.

(5) Optimization of Composite Ion Resin Performance Under Variable Conditions

Based on the results of static decolorization screening, one anion or cation resin that comprehensively satisfied the three metrics (decolorization rate, desalination rate, and xylan retention rate) was selected for compounding. The effects of the composite ion resin on xylan solution decolorization, desalination, and xylan retention were investigated under the following variable conditions: adsorption time (30, 60, 90, 120, and 150 min); anion-cation resin ratio (2:1, 1.5:1, 1:1, 1:1.5, and 1:2); adsorption temperature (30, 35, 40, 45, and 50 °C); and pH (3, 4, 5, 6, and 7).

(6) Post-Purification Processing of Xylan

Finally, the decolorized and desalted solution (treated with ion exchange resins) was added to anhydrous ethanol at a volume ≥ 3 times that of the solution and allowed to stand overnight for xylan precipitation. After distinct precipitation stratification was observed, filtration was performed. The solid precipitate was then placed in a fume hood for ≥ 6 h to allow ethanol volatilization, followed by freeze-drying to obtain xylan solids. After appropriate grinding, alkaline-extracted xylan powder from *Camellia oleifera* shell was obtained. Through this systematic purification process, moisture, ethanol solvent residues, and inorganic salt impurities in the xylan sample were effectively removed, ultimately yielding a refined xylan sample meeting high-purity grade requirements.

3.1.5 Fourier Transform Infrared (FT-IR) Spectroscopic Analysis of *Camellia oleifera* Shell Xylan Powder

Fourier transform infrared spectroscopy (FT-IR) was employed to collect infrared absorption spectra of the samples over a wavenumber range of 4000–500 cm^{-1}. The *Camellia oleifera* shell xylan powder was mixed with potassium bromide (KBr) at a mass ratio of 1:100, and the mixture was pressed into a transparent pellet using a tablet press for spectral measurement.

3.1.6 Electron Microscopy Analysis and Particle Size Determination of *Camellia oleifera* Shell Xylan Powder

The morphology of xylan was observed using an FEI Tecnai G2 F30 field-emission transmission electron microscope (FE-TEM) and a ZEISS GeminiSEM 300 scanning electron microscope (SEM, Germany). The particle size of xylan was measured and calculated based on the microscopic images.

3.1.7 Nuclear Magnetic Resonance (NMR) Analysis of *Camellia oleifera* Shell Xylan Powder

Proton nuclear magnetic resonance (^{1}H-NMR) and carbon-13 nuclear magnetic resonance (^{13}C-NMR) spectra of the xylan samples were acquired using a 600 MHz AVANCE III NMR spectrometer (Bruker Corporation, Germany). For sample preparation: 20 mg of xylan was precisely weighed and transferred to an NMR tube; 0.5 mL of deuterium oxide (D_2O) was added, and the mixture was vortexed until the sample was completely dissolved. Acquisition parameters were set as follows: ^{1}H-NMR: 600 MHz resonance frequency, 16 scans; ^{13}C-NMR: 150.91 MHz resonance frequency, 4096 scans, and a relaxation delay of 2 s.

3.1.8 Data Processing Method

The data processing method was consistent with that described in Section 1.1.8.

3.2 Results and Analysis

3.2.1 Effect of Different Extraction Conditions on Xylan Yield

Under fixed experimental conditions of a reaction time of 3 h, a temperature of 80 °C, and a solid-to-liquid ratio of 1:12 g/mL, this study investigated the influence of sodium hydroxide (NaOH) solution concentration (4%, 6%, 8%, 10%, 12%) on the yield of xylan extraction. The specific experimental results are detailed in Figure S11(A). As the NaOH concentration increased, the xylan extraction efficiency showed a significant trend of change. At the low concentration of 4%, the xylan extraction efficiency was at its lowest level. When the concentration was increased to 10%, the extraction efficiency reached its peak. This phenomenon is attributed to the fact that the 10% alkaline solution effectively promotes the expansion of lignocellulose, thereby achieving complete dissolution of xylan. When the NaOH concentration reached 12%, the xylan yield showed a downward trend. This is because a high concentration of alkaline solution not only significantly increases production costs but also leads to an increase in the salt content in the product, thereby negatively affecting the final product quality. Previous research has found that for producing films or packaging materials, hemicellulose extracted at lower alkali concentrations has a relatively higher molecular weight and possesses mechanical integrity even without adding plasticizers. Therefore, a NaOH solution concentration of 10% was selected as the optimal alkali concentration.

Under fixed experimental conditions of a reaction time of 3 h, a NaOH solution concentration of 10%, and a solid-to-liquid ratio of 1:12 g/mL, this study systematically investigated the influence of reaction temperature (70, 75, 80, 85, 90 °C) on the yield of xylan extraction. The specific experimental results are detailed in Figure S11(B). Elevated temperature increases molecular kinetic energy, accelerates penetration of the alkaline solution into the plant cell wall, and promotes cleavage of chemical bonds (such as ester bonds and ether bonds) between lignin and hemicellulose, thereby releasing xylan. Furthermore, the solubility of xylan in alkaline solutions increases with temperature, enhancing the extraction rate. However, as temperature gradually increases, lignin dissolution is also promoted, and pigment impurities in the system increase, affecting subsequent purification. As shown in Figure S11(B), the xylan yield was highest at 80 °C, approaching 25%. When the temperature exceeded 80 °C, the extraction rate did not increase significantly. At elevated temperatures, some xylan in the system may be converted into other sugar derivatives, causing the yield to decrease. Therefore, 80 °C was identified as the optimal temperature for xylan extraction.

Under fixed experimental conditions of a reaction time of 3 h, a NaOH solution concentration of 10%, and a reaction temperature of 80 °C, this study further investigated the influence of different solid-to-liquid ratios (1:8, 1:10, 1:12, 1:14, 1:16 g/mL) on the yield of xylan extraction. The specific experimental results are detailed in Figure S11(C). Sufficient liquid allows the raw material to fully swell, increases the surface area, and promotes penetration of the alkaline solution into the cell wall, thereby releasing xylan. At a solid-to-liquid ratio of 1:8, the amount of alkaline solution was too small, limiting immersion and reaction uniformity of the raw material, potentially leading to local high temperature or concentration, accelerating

xylan degradation or side reactions. Conversely, excessive liquid may dilute the alkaline concentration, affecting reaction efficiency and increasing subsequent processing costs. At a solid-to-liquid ratio of 1:12, the xylan yield reached its maximum value. Therefore, a solid-to-liquid ratio of 1:12 was determined as optimal for xylan extraction.

Under fixed conditions of a NaOH solution concentration of 10%, a reaction temperature of 80 °C, and a solid-to-liquid ratio of 1:12, the influence of different reaction times on the extraction efficiency of xylan was studied, as shown in Figure S11(D). In the initial stage of the reaction, as time increased, the dissolution rate of xylan was much higher than the degradation rate, and the concentration of xylan in the liquid phase rapidly increased, with a significant increase in yield. However, prolonged treatment promoted lignin dissolution and increased the concentration of impurities in the liquid phase. When the treatment duration exceeded 3 h, the extraction efficiency of xylan showed no significant upward trend. Based on the principle of energy conservation and consumption reduction, and considering both extraction efficiency and energy consumption, 3 h was determined as the optimal treatment duration.

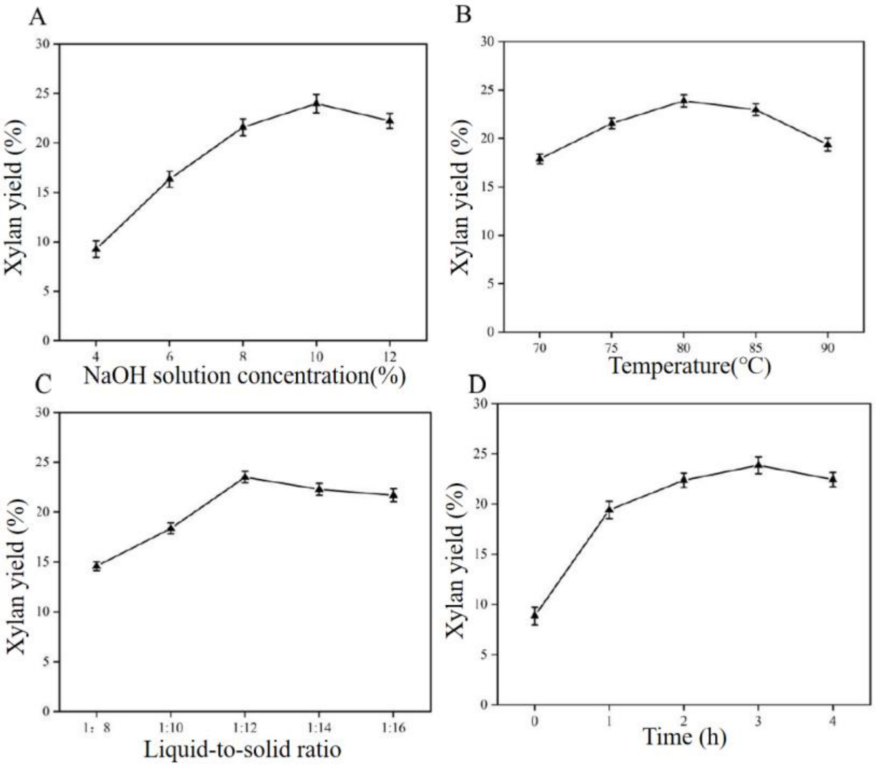

Fig. S11. Effect of different extraction conditions on xylan yield. (A) Effect of NaOH concentration on xylan yield; (B) Effect of temperature on xylan yield; (C) Effect of solid-liquid ratio on xylan yield; (D) Effect of reaction time on xylan yield.

3.2.2 Optimization of Process Parameters via Orthogonal Experimental Design

Building upon the findings of single-factor experiments, this study selected sodium hydroxide (NaOH) solution concentration (Factor A), reaction temperature (Factor B), solid-to-liquid ratio (Factor C), and reaction duration (Factor D) as key process parameters. A four-factor, three-level orthogonal experimental array $L_9(3^4)$ was constructed to systematically optimize the parameter combination for xylan preparation. Detailed experimental design and result analysis are presented in Table S5.

Table S5 Orthogonal experimental results for xylan extraction

Run Number	Factor A:Alkaline Solution Concentration(%)	B:Reaction Temperature (℃)	C:Solid-to-Liquid Ratio	D: Reaction Duration(h)	Xylan Extraction Yield (%)
1	8	75	1:12	2	17.89
2	8	80	1:14	3	19.86
3	8	85	1:16	4	19.52
4	10	75	1:14	4	21.37
5	10	80	1:16	2	24.22
6	10	85	1:12	3	22.86
7	12	75	1:16	3	20.44
8	12	80	1:12	4	21.87
9	12	85	1:14	2	17.34
K1	57.27	59.7	62.62	59.45	
K2	68.45	65.95	58.57	63.16	
K3	59.65	59.72	64.18	62.76	
k1	19.09	19.90	20.87	19.82	
k2	22.82	21.98	19.52	21.05	
k3	19.88	19.91	21.39	20.92	
R	3.73	2.08	1.35	1.24	
Factor Priority Ranking	A>B>C>D				
Optimal Condition Combination	A2	B2	C3	D2	

Based on the data in Table S5, the optimal process combination for xylan preparation was $A_2B_2C_3D_2$. The factor exerting the greatest influence on xylan yield was A (NaOH concentration). The order of influence of each factor on yield was A > B > C > D, with the range of factor A being the largest, making it the primary factor affecting xylan yield, while B, C, and D were secondary factors. The experimental results demonstrated that under the optimal process conditions of 10% NaOH solution concentration, 80 °C reaction temperature, 1:16 solid-to-liquid ratio, and 3 h reaction time, the xylan extraction efficiency reached a peak of 24.22%.

The confirmation result (24.22%, obtained at the predicted optimal combination $A_2B_2C_3D_2$: 10% NaOH, 80 °C, 1:16, 3 h) is now reported separately beneath the table. As now explained in the note below Table S5, factor D (reaction time) had the smallest range (R = 1.24) among the four factors, i.e., it was the least influential variable; accordingly, the yield obtained at 3 h (24.22%) coincided with that obtained at 2 h (24.22%), which is why the two measurements were identical.

3.2.3 Screening of Different Single Resins for Static Decolorization and Desalination of Xylan Solution

Ion exchange resins are high-molecular-weight polymers with functional groups and a network structure, carrying ion-exchange active groups, and are insoluble in acids, bases, and organic solvents. They offer advantages such as high adsorption capacity, broad processing range, and the ability to be regenerated and reused repeatedly, which aligns well with current food safety requirements [72]. The core function of ion exchange resins lies in their ability to undergo reversible and selective exchange reactions with ions of the same charge in solution, thereby achieving separation and purification of target ions. Figure S12 shows the effects of

different cation and anion exchange resins on the static decolorization and desalination of xylan solution.

The results indicate that 001×7 exhibited poor decolorization effect on xylan, and both cation exchange resins had relatively minor effects on decolorization, suggesting that decolorization efficacy is primarily influenced by anion exchange resins. Anion exchange resins can directly adsorb negatively charged pigment molecules through electrostatic interaction or chemical bonding to achieve decolorization. Among the anion exchange resins, both D301 and 717 achieved decolorization rates above 60%, with D301 demonstrating the best decolorization performance. Different ion exchange resins exhibited significant selective adsorption characteristics for ions present in solution. In the static adsorption and desalination experiments using four types of ion exchange resins, both types showed high desalination performance, with anion exchange resin D301 displaying superior overall performance in terms of salt removal rate and pigment clearance rate. After comprehensive evaluation, D301 resin achieved an optimal balance between desalination efficiency and decolorization performance and was therefore selected as the preferred ion exchange resin.

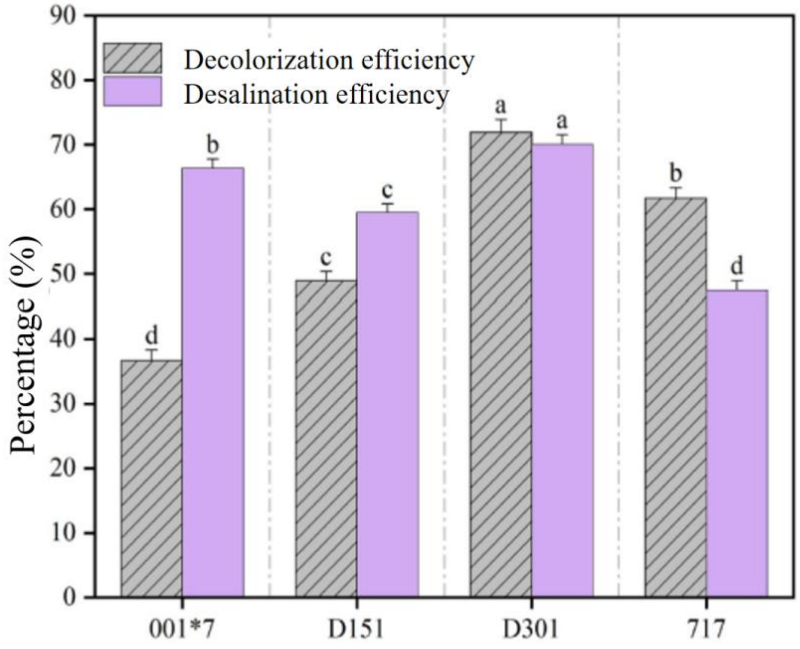

Fig. S12. Effect of different cation and anion exchange resins on static decolorization and desalination of xylan solution

3.2.4 Screening of Different Resin Combinations for Static Decolorization and Desalination of Xylan Solution

Ion-exchange resins exhibit selective adsorption capabilities for specific ions. If only anion or cation resins are used to treat the solution, it may lead to significant pH changes. Therefore, to maintain the acid-base balance of the solution and efficiently remove various ionic impurities, a series connection of cation and anion exchange resins for combined operation is commonly employed [73]. Figure S13 shows the effects of different 1:1 anion-cation resin combinations on the static decolorization and desalination of xylan solution.

The 001×7 + D301 and D151 + D301 resin combinations exhibited the best decolorization and desalination effects, achieving decolorization rates of 85.44% and 80.5%, and desalination rates of 75.7% and 72.56%, respectively. This is primarily attributed to the fact that chromophoric groups in pigments, such as –N=N– and –HC=CH–, typically carry negative charges, and the D301 anion resin possesses strong adsorption and exchange capacity for these groups. In the desalination and decolorization of sugar solutions, the D151 macroporous

weakly basic anion resin offers advantages in terms of easier handling and lower regenerant waste liquid costs compared to the 001×7 gel-type strongly basic anion resin. The D151 resin, with its macroporous structure, weakly basic functional groups, and anti-pollution characteristics, demonstrates superior comprehensive performance in sugar solution decolorization and desalination.

Figure S14 shows the effects of different anion-cation resin combinations on the retention rate of xylan. Compared to the other five resin combinations, the D151 + D301 resin combination achieved the highest xylan retention rate of approximately 80%. This is because the D151 resin, with its larger pore size and higher adsorption selectivity, exhibits a lower xylan loss rate in xylan solutions and is suitable for processing xylan extracts. Although the D301 resin has a slightly lower xylan retention rate due to its smaller pore size, its excellent decolorization capacity meets industrial requirements for xylan decolorization.

After considering multiple indicators including desalination performance, decolorization effect, and economic cost, this study determined that the anion resin D301 and cation resin D151 constitute the preferred ion-exchange resin combination. This combination demonstrates excellent synergy in salt removal, pigment removal, and cost control, effectively meeting the comprehensive requirements of the xylan purification process.

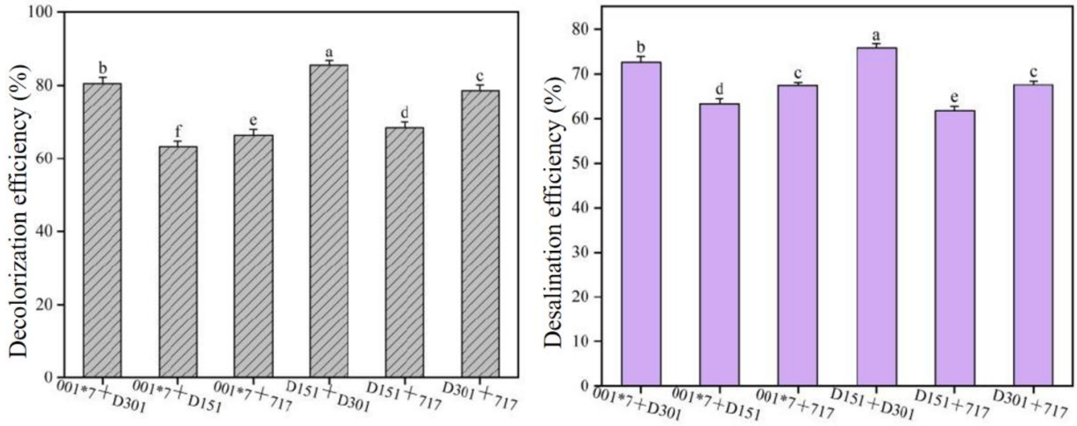

Fig. S13. Effect of different anion-cation resin combinations on static decolorization and desalination of xylan solution

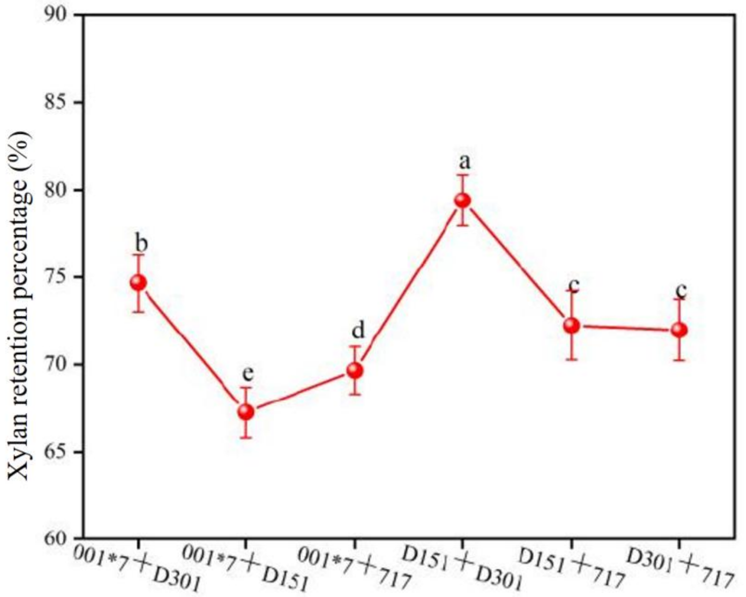

Fig. S14. Effect of different anion-cation resin combinations on xylan retention

3.2.5 Optimization of Decolorization and Purification Process Using D151 + D301 Composite Resin

(1) Influence of Temperature on the Decolorization and Purification Process of D151 + D301 Composite Resin

A 60 mL aliquot of crude xylan extract was taken and the pH was adjusted to 6.0. D151 ion exchange resin and D301 ion exchange resin were mixed in a 1:1 ratio (10 g:10 g) and added to each xylan solution. Static adsorption was carried out for 90 min. The effect of different reaction temperatures on the static adsorption decolorization and purification process of crude xylan solution was investigated. The results are shown in Figure S15.

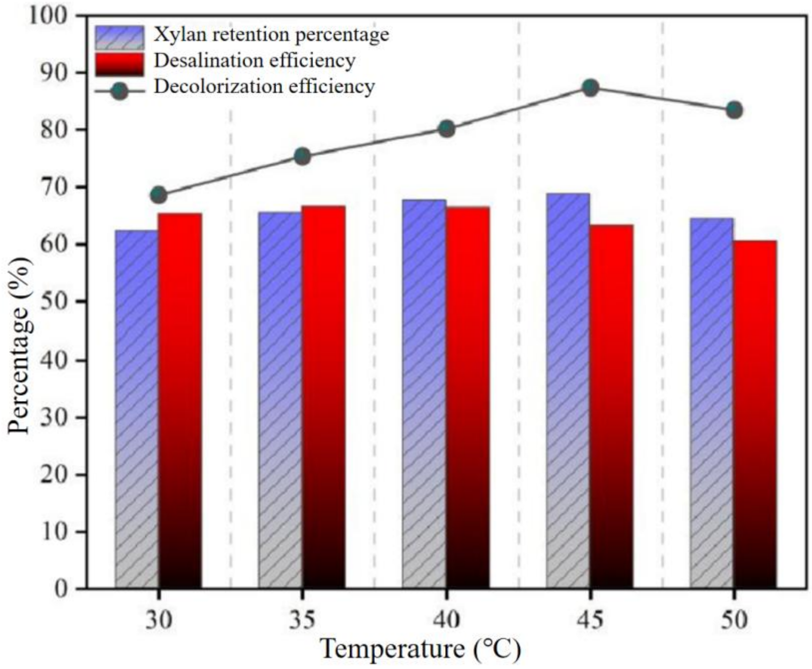

Fig. S15. Effect of different reaction temperatures on xylan decolorization, desalination, and xylan retention

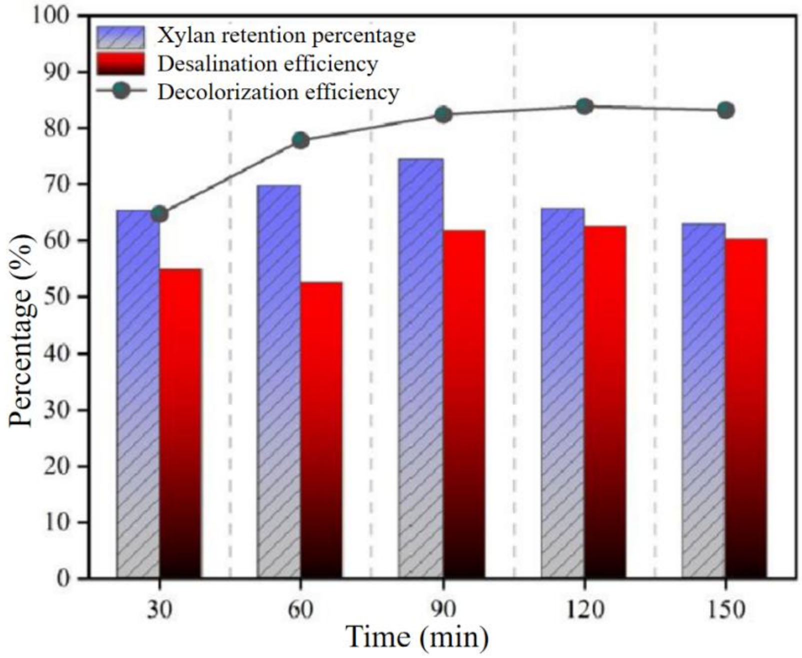

Fig. S16. Effect of different reaction times on xylan decolorization, desalination, and xylan retention

The experimental results indicate that temperature parameters have a limited influence on desalination efficiency, but exhibit a trend of initially promoting and subsequently inhibiting xylan retention. Compared with desalination rate and xylan retention rate, temperature exerts a more significant regulatory effect on decolorization efficiency. Under the optimized temperature condition of 45 °C, the peak decolorization rate reached 87.38%, with a desalination rate of 63.21% and a xylan retention rate of 68.77%, demonstrating the advantage of this temperature in balancing decolorization effectiveness with protection of heat-sensitive components. Further temperature increases resulted in a slight downward trend in both decolorization rate and xylan retention rate. This phenomenon can be attributed to disruption of the dynamic equilibrium between adsorption and desorption processes at elevated temperatures, causing previously adsorbed pigments to be re-released into solution, thereby reducing decolorization efficiency and affecting xylan retention levels. Therefore, 45 °C was adopted as the optimal decolorization temperature for the D151 + D301 composite resin system.

(2) Influence of Time on the Decolorization and Purification Process of D151 + D301 Composite Resin

A 60 mL aliquot of crude xylan extract was taken and the pH was adjusted to 6.0. D151 ion exchange resin and D301 ion exchange resin were mixed at a 1:1 ratio (10 g:10 g) and added to each xylan solution. The effect of different adsorption times on the static adsorption decolorization and purification process of the crude xylan solution was investigated at a reaction temperature of 45 °C. The results are shown in Figure S16.

With increasing time, the decolorization rate continuously rose. By 90 min, the decolorization rate had stabilized at approximately 80%, indicating that pigment adsorption by the ion exchange resins had reached equilibrium. The desalination rate exhibited a trend of initially decreasing, then increasing, and finally stabilizing. Conversely, the xylan retention rate showed the opposite pattern. Further extension of decolorization time beyond 90 min resulted in saturation of pigment adsorption by the D151 and D301 resins, but led to a significant decrease in xylan retention rate. Therefore, a decolorization time of 90 min was selected as optimal.

(3) Influence of Blend Ratio on the Decolorization Process of D151 + D301 Composite Resin

A 60 mL aliquot of crude xylan extract was taken and the pH was adjusted to 6.0. Under the conditions of a reaction temperature of 45 °C and a static adsorption time of 90 min, the effects of different blending ratios of D151 and D301 ion exchange resins on the static adsorption decolorization and purification process of the crude xylan solution were investigated. The results are shown in Figure S17.

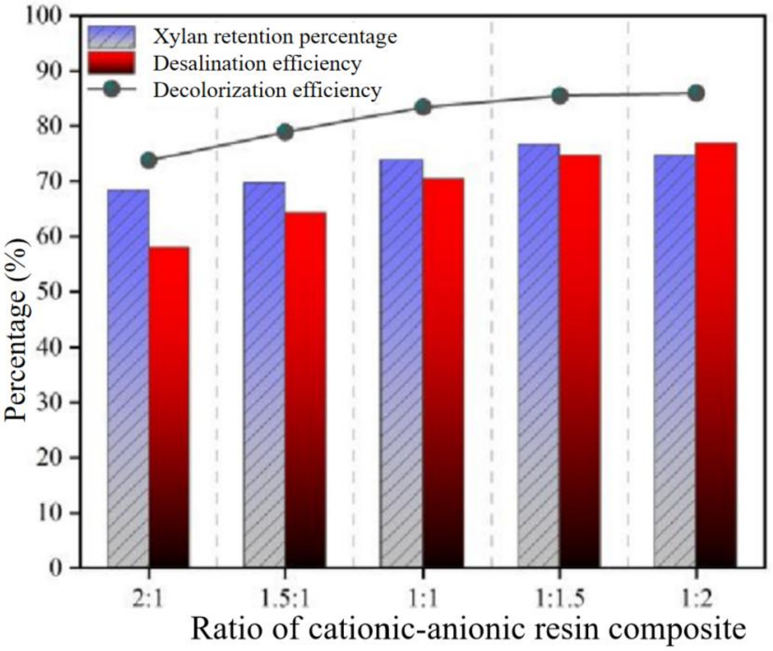

Fig. S17. Effect of different composite resin ratios on xylan decolorization, desalination, and xylan retention

As the ratio of cationic to anionic resins decreased, the decolorization rate gradually increased. This trend is attributed to the enhanced decolorization capacity of the anionic exchange resin D301 as its proportion in the system increased. When the ratio of cationic to anionic resins reached 1:1.5, the decolorization rate tended to stabilize. The desalination rate also exhibited an upward trend under these conditions. The retention rate of xylan initially increased and then decreased. This pattern can be explained by competitive adsorption: when D151 dominated the system, it preferentially adsorbed large molecular pigments, reducing pigment competition for D301 and consequently increasing xylan retention. As the proportion of D301 increased, desalination capacity improved synergistically; however, the increased proportion of small-pore resins raised the probability of physical entrapment of xylan molecules. At a cationic-to-anionic resin ratio of 1:1.5, the xylan retention rate reached its maximum of 76.59%, with corresponding decolorization and desalination rates of 85.49% and 74.69%, respectively. After comprehensive consideration of all three performance indicators, the optimal ratio of cationic exchange resin to anionic exchange resin was determined to be 1:1.5.

(4) Influence of pH on the Decolorization Process of D151 + D301 Composite Resin

A 60 mL aliquot of crude xylan extract was taken. D151 ion exchange resin and D301 ion exchange resin were mixed at a ratio of 1:1.5 (8 g:12 g) and added to each xylan solution. The effect of different adsorption pH values on the static adsorption decolorization and purification process of crude xylan solution was investigated at a reaction temperature of 45 °C. The results are shown in Figure S18.

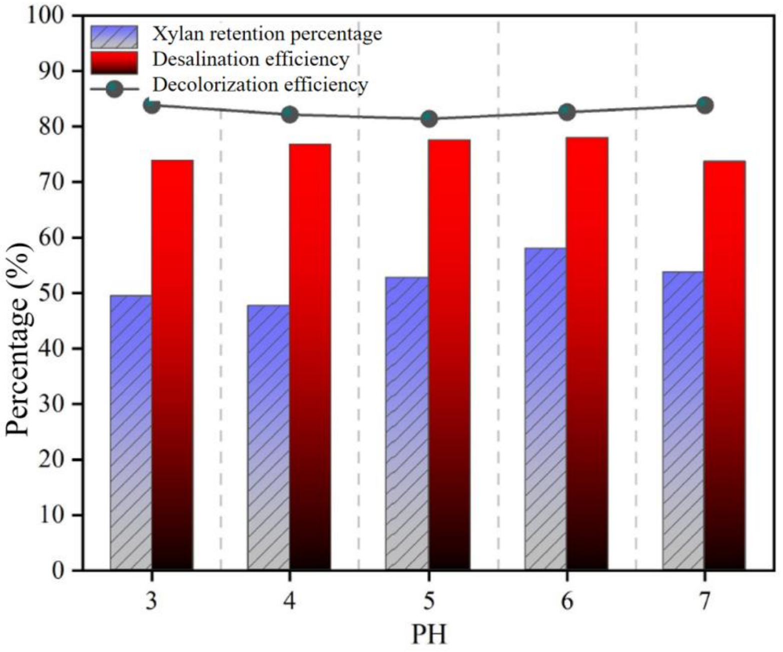

Fig. S18. Effect of different reaction pH on xylan decolorization, desalination, and xylan retention

As the pH value gradually increased, the decolorization rate and xylan retention rate exhibited a pattern of initially decreasing and then increasing, while the desalination rate first increased and then decreased. In acidic environments, the composite resin demonstrated enhanced adsorption of pigments from solution. Conversely, under alkaline conditions, the xylan loss rate increased correspondingly. As shown in the figure, the regulatory effect of solution pH on decolorization efficiency, desalination efficiency, and xylan retention efficiency was relatively limited within the tested range. When the system pH was adjusted to 6.0, all three technical indicators reached optimal levels, indicating that this pH condition is conducive to maintaining the synergistic balance among decolorization, desalination, and xylan retention.

Under the optimized decolorization and desalination process parameters—temperature of 45 °C, reaction time of 90 min, cationic-to-anionic resin ratio of 1:1.5, and system pH of 6.0—the best treatment performance was achieved. Under these conditions, the decolorization rate reached 85.7%, the desalination rate attained 67.63%, and the xylan retention efficiency remained at 73.45%, fully validating the synergistic optimization effect of the selected process parameters.

3.2.6 Xylan Analysis and Characterization

(1) Fourier Transform Infrared Spectroscopy Analysis

As shown in Figure S19, the infrared spectrum of xylan exhibited a broad peak at 3295 cm^{-1} corresponding to the stretching vibration of hydroxyl groups (–OH). The structure of xylan hemicellulose contains numerous hydroxyl groups, thereby contributing to its hydrophilic character. The absorption peak observed at 2920 cm^{-1} is attributed to the stretching vibration of C–H functional groups. The absorption peak at 1066 cm^{-1} serves as a significant marker of the inherent characteristics of xylose molecules, representing the stretching vibration of the ether bond (C–O–C). The absorption peak at 902 cm^{-1} is specifically assigned to the unique configuration of β-D-xylose, indicating that the sugar units are linked through β-glycosidic bonds. The region spanning 1170–1000 cm^{-1} contains characteristic peaks of the arabinoxylan backbone, confirming that the primary component of the extracted sample is xylan. The absorption peak at 1162 cm^{-1} indicates the presence of arabinose side groups. Based on the infrared spectral analysis, it can be concluded that the xylan extracted from *Camellia oleifera*

shells is composed predominantly of xylose. These characteristic peaks are consistent with the infrared features reported for corn cob xylan and for xylan extracted via ultrasonic-assisted low-temperature alkaline extraction [74].

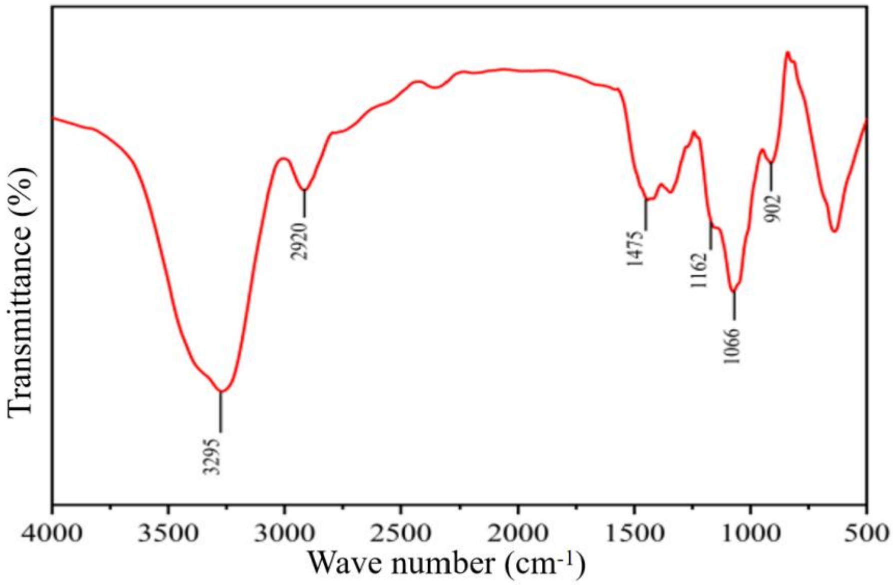

Fig. S19. FT-IR spectrum of xylan

(2) Electron Microscopy Analysis

Due to their high surface energy, the xylan particles tended to aggregate, indicating the occurrence of agglomeration [75]. The particle size distribution of xylan was calculated through SEM image analysis. As shown in Figure S20, 80% of the xylan particles fell within the size range of 0.5–2 μm, with the largest proportion (29.42%) distributed in the 1–1.5 μm range. The presence of low-micron-sized xylan particles in blend systems can form tortuous barrier pathways, thereby potentially improving barrier performance against water vapor or gas transmission.

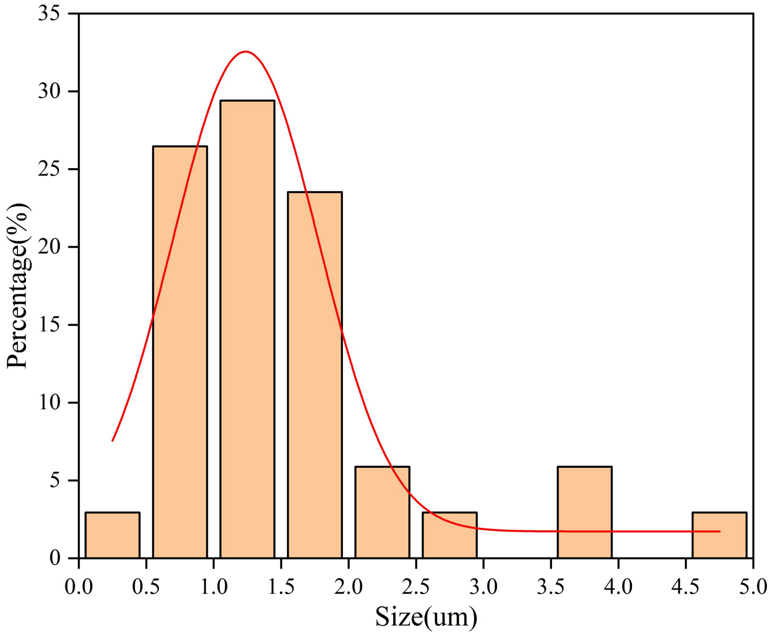

Fig. S20. Particle size distribution of xylan

(3) Nuclear Magnetic Resonance Analysis

To elucidate the chemical structure characteristics of alkali-extracted xylan, this study employed nuclear magnetic resonance spectroscopy for precise molecular structure characterization, with particular focus on the main chain configuration and side chain structure. Figures S21 and S22 present the ^{1}H-NMR and ^{13}C-NMR spectra of alkali-extracted xylan, respectively. Signal assignments were made based on relevant literature.

Figure S21 shows the ^{1}H-NMR spectrum of the xylan sample. The anomeric proton signals of α-configured sugars typically appear at δ 5.1–5.8 ppm, while those of β-configured sugars resonate at δ 4.4–4.8 ppm. The peaks at δ_h 4.36, 3.53, 3.46, 3.35, 3.33, and 4.08 ppm were assigned to the H-1, H-2, H-3, H-4, H-5a, and H-5b absorption peaks of the β-D-xylopyranose unit, respectively. The peak at δ_h 1.20 ppm was attributed to the H-6 absorption of rhamnose residues, and the strong signal at δ_h 4.73 ppm was assigned to residual deuterium oxide (D_2O) solvent.

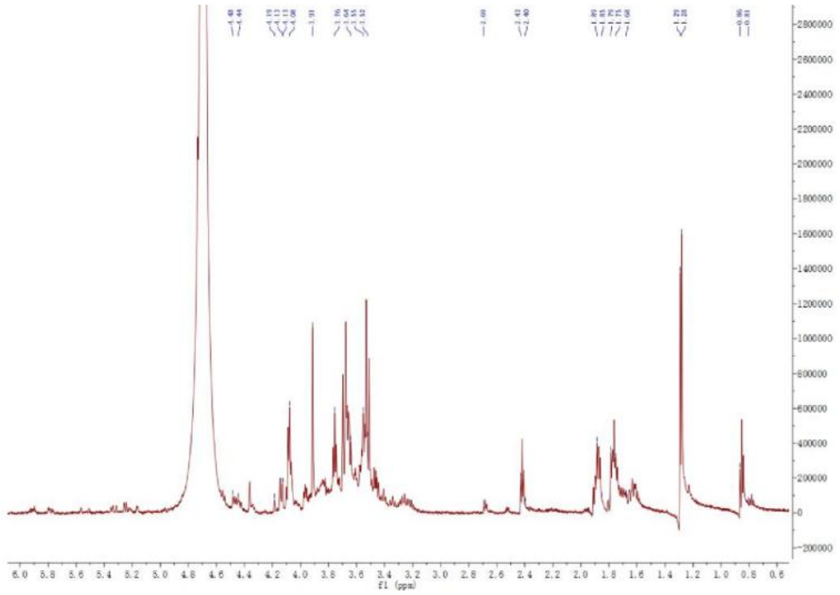

Fig. S21. ^{1}H-NMR spectrum of xylan from *Camellia oleifera* husks

For the ^{13}C-NMR spectrum, the anomeric carbons of polysaccharides predominantly appear in the region of δ 92–110 ppm, while ring carbons (C2–C5) typically resonate in the 55–85 ppm range. Signals in the 58–68 ppm region are assigned to C6 (pyranose form) or C5 (furanose form) methylene carbons (–CH_2–) outside the sugar ring. Multiple signals in the 68–85 ppm range correspond to the ring carbons C2–C4. Significant signal overlap in this region is consistent with the characteristic complexity of polysaccharide carbon spectra. Figure S22 displays the ^{13}C-NMR spectrum of this polysaccharide. The chemical shifts at δ_c 105.91, 78.29, 75.10, 76.50, and 65.78 ppm were assigned to the C-1, C-2, C-3, C-4, and C-5 positions of the arabinose side chains attached to the β-D-xylose backbone, respectively.

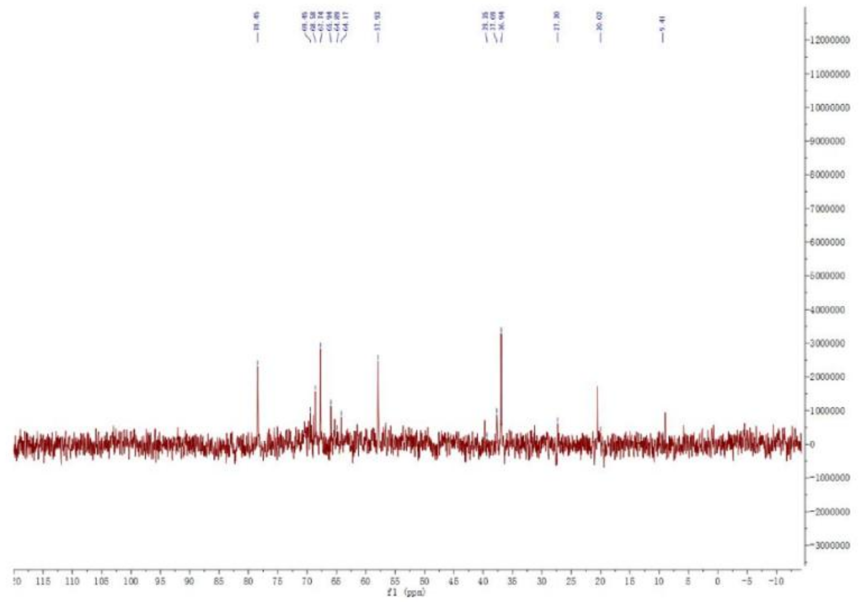

Fig. S22. ^{13}C-NMR spectrum of xylan from *Camellia oleifera* husks

4.The strawberry sampling scheme for each treatment group

Table S6. The strawberry sampling scheme for each treatment group (60 fruit per treatment).

Storage time (day)	Fruit for decay incidence (4-fruit package)	Fruit for six quality indices (6-fruit package)	Sub-total per treatment
0	4	6	10
2	4	6	10
4	4	6	10
6	4	6	10
8	4	6	10
10	4	6	10
Total per treatment	24	36	60

Four treatments (CK, PE, AX/PVA, 6% TS/AX/PVA) × 60 = 240 strawberries in total.

With respect to experimental units and packaging: at each storage time, each treatment used two independent packages — one package containing 4 fruit (the experimental unit for decay incidence) and one package containing 6 fruit (the experimental unit for the six quality indices). Because the preservation mechanism relies on a semi-enclosed headspace, the package (rather than the individual fruit) constitutes the experimental unit for the packaging treatment; individual fruit within a package were treated as sub-samples. We also explicitly state in the revised Methods that one-way ANOVA followed by Duncan's multiple-range test was performed separately at each storage time, and we acknowledge that this approach does not model or test treatment × time interactions, which is stated as a statistical limitation.

Each experimental unit consisted of strawberries arranged on a small plastic tray and sealed with the corresponding film (PE or TS/AX/PVA); the CK (unsealed control) was placed on the same tray type without a covering film. Four fruit were packaged together on one tray for the decay-incidence assessment, and six fruit were packaged together on one tray for the six quality indices. The film was applied over the tray without contacting the fruit surface,

leaving a semi-enclosed headspace between the film and the fruit; no gas flushing was applied, so the headspace initially contained ambient air and gas exchange with the external environment occurred only through the film. Films were opened and discarded after each destructive measurement.

5. Effect of AX/PVA volume ratio on tensile strength & elongation at break.

Table S7. Effect of AX/PVA volume ratio on tensile strength & elongation at break.

Volume ratio of AX to PVA	Tensile Strength (MPa)	Elongation at Break (%)
1:9	14.24	285.45±2.07
2:8	19.73	273.45±3.42
3:7	17.58	231.88±3.54
4:6	8.37	207.69±2.89
5:5	4.68	138.42±4.51
